# Tandem aldehyde–alkyne–amine coupling/cycloisomerization: A new synthesis of coumarins

**DOI:** 10.3762/bjoc.9.21

**Published:** 2013-01-28

**Authors:** Maddi Sridhar Reddy, Nuligonda Thirupathi, Madala Haribabu

**Affiliations:** 1 Medicinal & Process Chemistry Division, CSIR-Central Drug Research Institute, Lucknow-226 001, India, Fax: +91-(522)-2623405, Tel: +91–(522)–2612 411, Extn: 4379

**Keywords:** A^3^ coupling, cooperative catalysis, coumarin synthesis, cycloisomerization, transition-metal catalysts

## Abstract

Cu-catalyzed A^3^ coupling of ethoxyacetylene, pyrrolidine and salicylaldehydes led to a concomitant cycloisomerization followed by hydrolysis of the resultant vinyl ether to afford coumarins in a cascade process. The reaction proceeded through exclusive 6-*endo*-*dig* cyclization and is compatible with halo and keto groups giving coumarins in good to moderate yields.

## Introduction

An alkyne, an aldehyde and an amine coupling, referred to as A^3^ coupling [1], has been found as an efficient method for C–N and C–C bond formation that results in equivalence of reductive alkylation of amines while at the same time appending an alkyne, i.e., a highly useful moiety for further functionlization. This three-component coupling has been accomplished with a very broad range of transition metals, including copper, silver, gold, ruthenium/copper, cobalt, iridium and iron. Similarly, cycloisomerization of alkynols and alkynamines has also been an attractive approach for the synthesis of various known and new heterocyclic frameworks [2–22]. Various alkynophilic catalysts such as transition-metal catalysts (based on gold, mercury, platinum, silver, etc.), Brønsted acids and electrophilic iodine sources (I_2_, ICl, NIS) have been used for the transformation.

**Scheme 1:**
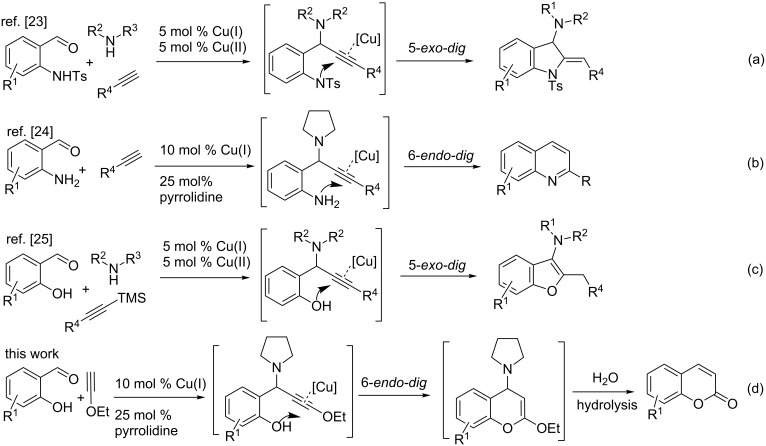
Synthesis of various heterocycles by a tandem A^3^ coupling/cycloisomerization strategy.

If one of the partners in A^3^ coupling has any nucleophile for concomitant electrophilic cyclization on the alkyne group in the A^3^ product, this may result in an interesting reaction sequence to produce various heterocycles. Recently, Gevorgyan and co-workers [23] used these two processes (A^3^/5-*exo*-*dig* cycloisomerization) in tandem to obtain indolines, which were then converted to useful substituted indole derivatives (Scheme 1, (a)). Similarly, Patil and Raut [24] reported an elegant method for the synthesis of 2-substituted quinolines from 2-aminobenzaldehydes and terminal alkynes by a tandem A^3^/6-*endo*-*dig*-cycloisomerization (Scheme 1, (b)) using a cooperative catalytic system consisting of CuI and pyrrolidine. Prior to these two findings, Sakai et al. [25] reported a facile synthesis of 3-aminobenzofurans through an A^3^ coupling and an exclusive 5-*exo*-*dig*-cycloisomerization (Scheme 1, (c)). Similarly, Yan and Liu [26], Fujii et al. [27–28], Chernyk and Gevorgyan [29], Ji et al. [30], and Wu et al. [31] reported the synthesis of aminoindolizines, 2-(aminomethyl)indoles, imidazopyridines, butenolides and 1,2-dihydroisoquinoline derivatives, respectively, combining these two approaches successfully. Along the same lines, we investigated a reaction between ethoxyacetylene, pyrrolidine and salicylaldehyde in the presence of a transition-metal catalyst. That, after consecutive A^3^ coupling, cycloisomerization and hydrolysis of the resultant vinyl ether intermediate, should produce coumarins (Scheme 1, (d)). The reason for the selective 6-*endo*-*dig* cyclization of such a cooperative-catalysis reaction has been well documented through DFT computational studies by Patil et al. in their recent publication [32].

## Results and Discussion

Coumarins [33–46] have been attractive targets [47–53] for synthetic chemists due to their frequent occurrence in nature and for their interesting biological and pharmaceutical applications. In continuation of our interest in the cycloisomerization of alkynols and alkynamines for the synthesis of various heterocycles [17–22], we herein report the synthesis of coumarins from salicylaldehydes by a Cu-catalyzed exclusive 6-*endo*-*dig* electrophilic cyclization of the intermediate hydroxyphenylpropargylamine as shown in Scheme 1 (d). We initially investigated the reaction with various Cu-, Au- and Pd-based catalysts in the presence of pyrrolidine in MeCN at room temperature (Table 1).

**Table 1:** Catalyst and condition screening.

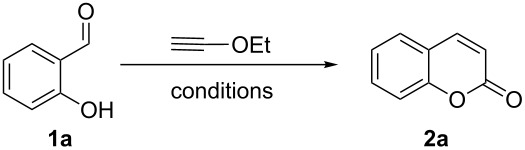					

entry	catalyst	solvent/temp	base	Time (h)	yield (%)

1	CuI	CH_3_CN/rt	pyrrolidine	24	58
2	Cu(OTf)_2_	CH_3_CN/rt	pyrrolidine	24	24
3	AuCl	CH_3_CN/rt	pyrrolidine	24	35
4	AuCl_3_	CH_3_CN/rt	pyrrolidine	24	48
5	HAuCl_4_	CH_3_CN/rt	pyrrolidine	24	50
6	PPh_3_AuCl	CH_3_CN/rt	pyrrolidine	24	30
7	PdCl_2_	CH_3_CN/rt	pyrrolidine	24	25
8	CuI	CH_3_CN/100 °C	pyrrolidine	2	65
9	—	CH_3_CN/100 °C	pyrrolidine	3	—
10	CuI	CH_3_CN/100 °C	pyrrolidine	3	—

The required product was obtained but in very low yield, and the reaction time was prolonged to more than 24 h. When the reaction temperature was raised to 100 °C in the presence of CuI and pyrrolidine in CH_3_CN, the desired product was obtained in 65% in 2 h.

Encouraged by this promising result, the scope of the reaction was tested with a number of salicylaldehydes. As is apparent from Table 2, the reaction is highly versatile, working efficiently with both electron-rich and -poor substrates. Substrates **1b–h** with various alkyl substituents produced the corresponding coumarins **2b–h** in 50–82% yield.

**Table 2:** Synthesis of coumarins **2** from salicylaldehydes **1** by A^3^ coupling/cycloisomerization.

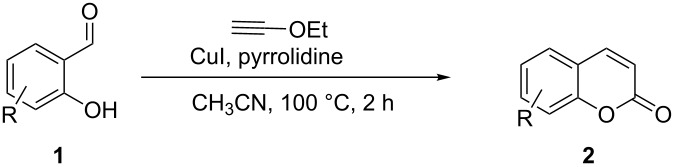							

entry	substrate **1**^a^	product **2**	yield (%)^b^	entry	substrate **1**^a^	product **2**	yield (%)^b^

1	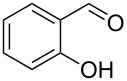 **1a**	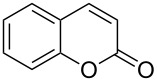 **2a**	62	9	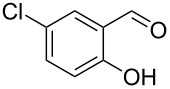 **1i**	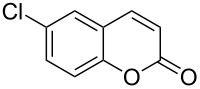 **2i**	62
2	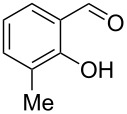 **1b**	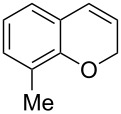 **2b**	68	10	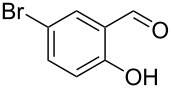 **1j**	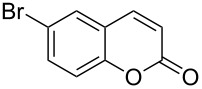 **2j**	62
3	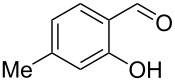 **1c**	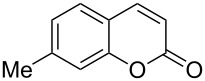 **2c**	78	11	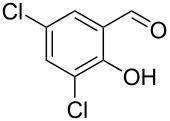 **1k**	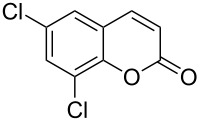 **2k**	60
4	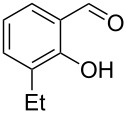 **1d**	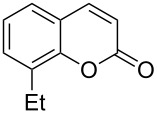 **2d**	75	12	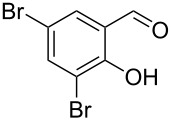 **1l**	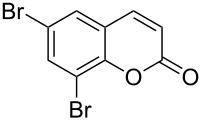 **2l**	65
5	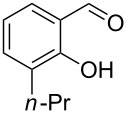 **1e**	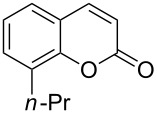 **2e**	80	13	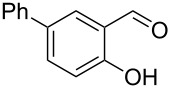 **1m**	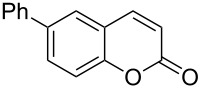 **2m**	76
6	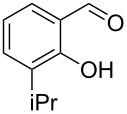 **1f**	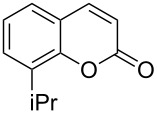 **2f**	82	14	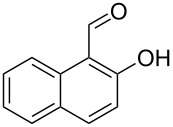 **1n**	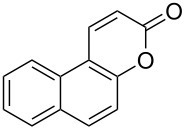 **2n**	65
7	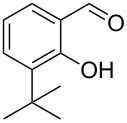 **1g**	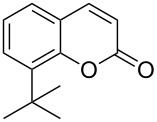 **2g**	50	15	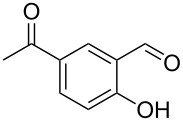 **1o**	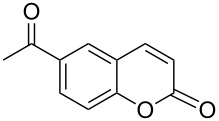 **2o**	84
8	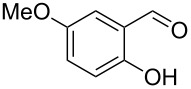 **1h**	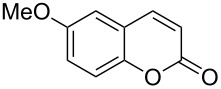 **2h**	80				

^a^All reactions were conducted with 1 mmol substrate in 0.25 M concentration. ^b^Isolated yields.

A slight reduction in yield was observed in the cases of halogen containing substrates. Thus, substrates **1i–l** gave the required products **2i–l** in 62–65% yield. Substrates **1m** and **1n** with extended conjugation also reacted well under the standardized conditions to give the corresponding products **2m** and **2n** in good yields (65–76%). The reaction is highly compatible with keto functionality, as is evident from the conversion of **1o** to **2o** in 84% yield. It should be noted that the reaction is limited to aldehydes and not to ketones, which do not undergo A^3^ coupling.

**Scheme 2:**
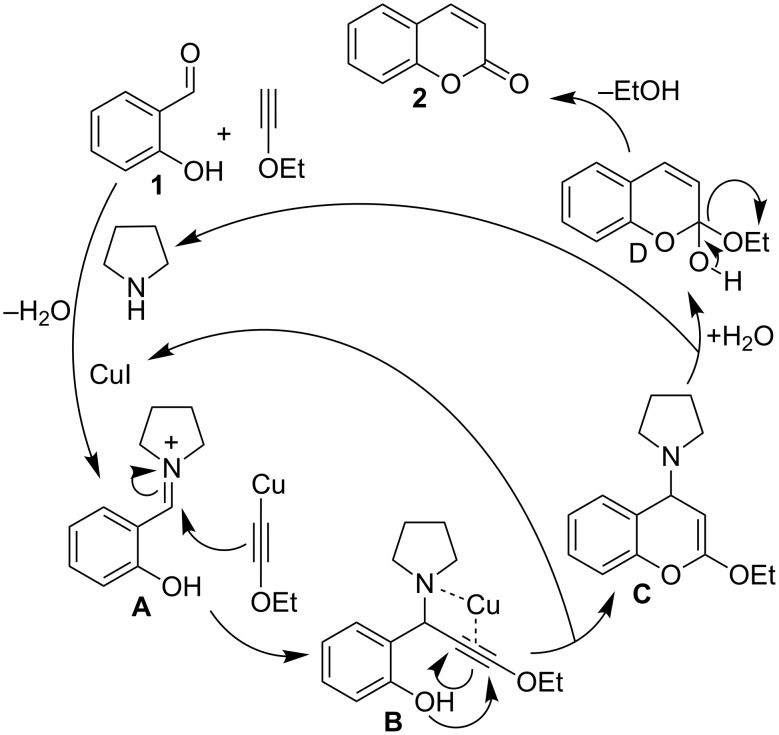
A plausible mechanistic pathway.

A plausible mechanism via a cooperative catalysis by Cu and pyrrolidine is described in Scheme 2 (with the assistance of the work reported by Patil et al. [24,32]). Initial condensation of pyrrolidine with salicylaldehyde **1** produced iminium intermediate **A**. The addition of copper ethoxyacetylide, formed on the reaction of ethoxyacetylene with Cu, to the iminium intermediate **A** yielded propargylamine intermediate **B**. Copper being coordinated with the amine group immediately activated the alkyne group to facilitate cycloisomerization with the phenoxy group, to produce vinyl ether **C**, which, being susceptible to hydrolysis, underwent water addition followed by an extrusion of the pyrrolidine molecule for further catalysis. The resulted intermediate **D** lost an EtOH molecule to furnish the required product **2**.

## Conclusion

In summary, a facile synthesis of coumarins is reported from readily available starting materials, i.e., salicylaldehydes and ethoxyacetylene, through a tandem A^3^ coupling and cycloisomerization cascade. The reaction was catalyzed by a pyrrolidine and copper iodide cooperative catalytic system, and the reaction was not observed in the absence of either of the catalysts. The yields are good to moderate and the reaction has a good substrate scope being compatible with halogen and keto groups. The process constitutes an easy and efficient access to highly valuable building blocks of natural products or biologically active compounds.

## Supporting Information

File 1 Experimental procedures and product characterization for compounds **2a–o**.
