# Supplementary material for: Tandem aldehyde–alkyne–amine coupling/cycloisomerization: A new synthesis of coumarins
Source: Beilstein J Org Chem. 2013 Jan 28;9:180–4. doi: 10.3762/bjoc.9.21 (PMC3566765; doi:10.3762/bjoc.9.21)
Supplement: File 1 — Experimental procedures and product characterization for compounds 2a–o. [file Beilstein_J_Org_Chem-09-180-s001.pdf]

Supporting Information

for

Tandem aldehyde–alkyne–amine coupling/cycloisomerization: A new synthesis of coumarins

Maddi Sridhar Reddy*, Nuligonda Thirupathi and Madala Haribabu

Address: Medicinal & Process Chemistry Division, CSIR-Central Drug Research Institute, Lucknow-226 001, India, Fax: +91-(522)-2623405, Tel: +91–(522)–2612 411, Extn: 4379

Email: Maddi Sridhar Reddy - msreddy@cdri.res.in

*Corresponding author

Experimental procedures and product characterization for compounds **2a–o**.

General Information …………………………………………………… S2

Experimental procedures and data of the compounds ………………… S2–S7

References ………………………………………………………………… S8

Copies of ^{1}H and ^{13}C NMR spectra …………………………………… S9–S37

General Information. All reagents were commercial and were used without further purification unless otherwise noted. Infrared spectra were recorded with FTIR as a thin film and are expressed in cm^{-1}. ^{1}H NMR (200 or 300 MHz) and ^{13}C NMR (50 or 75 MHz) spectra were recorded by using $CDCl_3$/DMSO as solvents and TMS as internal standard. Mass spectra were obtained on an ESI mass spectrometer and HR/ESI mass spectra were obtained on high resolution ESI mass spectrometer.

Experimental Section:

General Procedure for the synthesis of Coumarins [1]:

To a screw-cap vial containing a stir bar, 122 mg (1 mmol) of salicylaldehyde, 0.35 mL of ethoxyacetylene (40% by weight in hexanes, ca. 2 mmol), CuI (10 mol %), CH_3CN (2 ml) and pyrrolidine (25 mol %) were added. The reaction vial was fitted with a cap and heated at 100 °C for 2 h. The reaction mixture was allowed to warm to ambient temperature. The reaction mixture was diluted with ethyl acetate and filtered through a plug of silica gel. The filtrate was concentrated under reduced pressure and the resulting residue was purified by column chromatography (8–10% EtOAc in hexanes) to obtain pure **2a** in 62% yield (0.090 g).

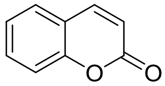

2*H*-Chromen-2-one (2a) [2]: (using general procedure, 94 mg of **2a** was obtained from 122 mg (1 mmol of **1a**) 65% yield; light brown solid, mp 81–83 °C; R_f = 0.4 (EtOAc/hexanes = 4:6); ^{1}H NMR (300 MHz, $CDCl_3$) δ: 7.7 (d, 1H, J = 9.4 Hz); 7.56 (d, 1H, J = 2.9 Hz); 7.51 (t, 1H, J = 7.8 Hz); 7.3 (d, 1H, J = 2.9 Hz); 7.27 (t, 1H, J = 2.9 Hz); 6.43 (d, 1H, J = 9.6 Hz); ^{13}C NMR (50 MHz, $CDCl_3$) δ:160.7, 154.0, 143.5, 131.8, 127.9, 124.4, 118.8, 116.8, 116.6; IR (KBr) ν: 2924, 1709, 1449, 1105, 759; ESIMS *m*/*z* $[M + H]^+$: 147.2; HRMS (ESI) m/z calcd for $C_9H_6O_2$ $[M + H]^+$: 147.0401, found:147.0456.

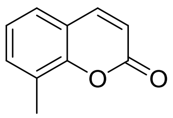

8-Methyl-2*H*-chromen-2-one (2b) [3]: (using general procedure, 108 mg of **2b** was obtained from 136 mg (1 mmol) of **1b**) 68% yield; white solid, mp 111–113 °C; R_f = 0.5 (EtOAc/hexanes = 3:7); ^{1}H NMR (300 MHz, $CDCl_3$) δ: 7.6 (d, 1H, *J* = 9.5 Hz); 7.37 (d, 1H, *J* = 6.9 Hz); 7.3 (d, 1H, *J* = 7.3 Hz); 7.1 (t, 1H, *J* = 7.3 Hz); 6.4 (d, 1H, *J* = 9.5 Hz); 2.4 (s, 3H); ^{13}C NMR (75 MHz, $CDCl_3$) δ: 160.9, 152.4, 143.7, 133.1, 126.3, 125.5, 123.9, 118.5, 116.2, 15.3.

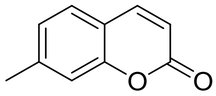

7-Methyl-2*H*-chromen-2-one (2c) [4]: (using general procedure, 124 mg of **2c** was obtained from 136 mg (1 mmol of **1c**) 78% yield; white solid, mp 165–167 °C; R_f = 0.5 (EtOAc/hexanes = 3:7); ^{1}H NMR (300 MHz, $CDCl_3$) δ: 7.6 (d, 1H, *J* = 9.6 Hz); 7.3 (d, 1H, *J* = 7.8 Hz); 7.1 (s, 1H); 7.0 (d, 1H, *J* = 7.8 Hz); 6.3 (d, 1H, *J* = 9.6 Hz); 2.4 (s, 3H); ^{13}C NMR (50 MHz, $CDCl_3$) δ: 161.1, 154.1, 143.4, 143.0, 127.5, 125.6, 116.9, 116.4, 115.3, 21.7.

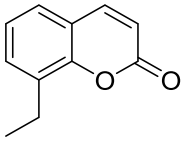

8-Ethyl-2*H*-chromen-2-one (2d): (using general procedure, 130 mg of **2d** was obtained from 150 mg (1 mmol of **1d**) 75% yield; light yellow oil; R_f = 0.4 (EtOAc/hexanes =2:8); ^{1}H NMR (300 MHz, $CDCl_3$) δ: 7.7 (d, 1H, *J* = 9.5 Hz); 7.4 (d, 1H, *J* = 7.5 Hz); 7.3 (d, 1H, *J* = 6.8 Hz); 7.2 (t, 1H, *J* = 7.5 Hz); 6.4 (d, 1H, *J* = 9.5 Hz); 2.9 (q, 2H, *J* = 7.5 Hz); 1.2 (t, 3H, *J* = 7.5 Hz); ^{13}C NMR (50 MHz, $CDCl_3$) δ: 161.0, 151.8, 144.0, 132.1, 131.6, 125.6, 124.2, 118.5, 116.1, 22.4,14.1.

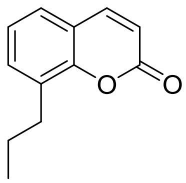

8-Propyl-2*H*-chromen-2-one (2e): (using general procedure, 150 mg of **2e** was obtained from 164 mg (1 mmol of **1e**) 80% yield; colorless oil; R_f = 0.5 (EtOAc/hexanes = 2:8); ^{1}H NMR (300 MHz, $CDCl_3$) δ: 7.7 (d, 1H, J = 9.7 Hz); 7.3 (d, 1H, J = 7.6 Hz); 7.3 (d, 1H, J = 7.1 Hz); 7.2 (t, 1H, J = 7.6 Hz); 6.4 (d, 1H, J = 9.7 Hz); 2.8 (t, 2H, J = 7.3 Hz); 1.7 (m, 2H); 0.9 (t, 3H, J = 7.3 Hz); ^{13}C NMR (50 MHz, $CDCl_3$) δ: 160.9, 151.9, 144.0, 132.4, 130.5, 125.7, 124.0, 118.6, 116.0, 31.1, 22.8, 13.8.

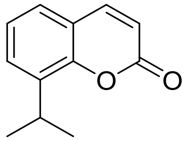

8-Isopropyl-2*H*-chromen-2-one (2f): (using general procedure, 154 mg of **2f** was obtained from 164 mg (1 mmol of **1f**) 82% yield; Colorless oil; R_f = 0.5 (EtOAc/hexanes = 3:7); ^{1}H NMR (300 MHz, $CDCl_3$) δ: 7.7 (d, 1H, J = 9.5 Hz); 7.4 (d, 1H, J = 7.3 Hz); 7.3 (d, 1H, J = 6.4 Hz); 7.2 (t, 1H, J = 7.3 Hz); 6.4 (d, 1H, J = 9.5 Hz); 3.6 (m, 1H); 1.3 (d, 6H, J = 6.9 Hz); ^{13}C NMR (75 MHz, $CDCl_3$) δ: 160.8, 151.3, 144.0, 136.5, 128.9, 125.5, 124.2, 118.6, 116.0, 26.4, 22.5.

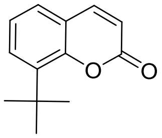

8-*tert*-Butyl-2*H*-chromen-2-one (2g) [3]: (using general procedure, 101 mg of **2g** was obtained from 202 mg (1 mmol of **1g**) 50 % yield; brown oil; R_f = 0.4 (EtOAc/hexanes = 2:8); ^{1}H NMR (300 MHz, $CDCl_3$) δ: 7.6 (d, 1H, J = 9.7 Hz); 7.5 (d, 1H, J = 7.6 Hz); 7.3 (d, 1H, J = 7.6 Hz); 7.2 (t, 1H, J = 7.5 Hz); 6.4 (d, 1H, J = 9.7 Hz); 1.5 (s, 9H); ^{13}C NMR (75 MHz, DMSO-d_6) δ: 159.4, 152.1, 145.1, 136.6 , 129.1, 126.9, 124.0, 119.1, 115.2, 34.4, 29.4; IR (KBr) ν: 2926, 1640, 1219, 770, 671; ESIMS m/z $[M + H]^+$: 203.2; HRMS (ESI) m/z calcd for $C_{13}H_{14}O_2$ $[M + H]^+$: 203.1027, found: 203.1036.

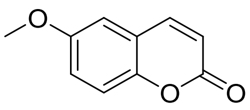

6-Methoxy-2*H*-chromen-2-one (2h) [5]: (using general procedure, 149 mg of **2h** was obtained from 152 mg (1 mmol of **1h**) 85% yield; brown oil; R_f = 0.3 (EtOAc/hexanes = 2:8); ^{1}H NMR (300 MHz CDCl$_3$) δ: 7.6 (d, 1H, *J* = 9.5 Hz); 7.2 (d, 1H, *J* = 2.7 Hz); 7.1 (dd, 1H, *J* = 9.04, 2.7 Hz); 6.9 (d, 1H, *J* = 2.7 Hz); 6.4 (d, 1H, *J* = 9.5 Hz); 3.8 (s, 3H); ^{13}C NMR (75 MHz, DMSO-d_6) δ: 160.0, 155.5, 147.8, 143.9, 119.3, 119.1, 117.2, 116.5, 110.6, 55.6; IR (KBr) ν: ESIMS m/z [M + H]$^+$: 177.1; HRMS (ESI) *m*/*z* calcd for $C_{10}H_8O_3$ [M + H]$^+$: 177.0507, found: 177.0536.

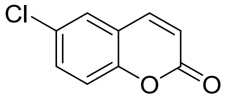

6-Chloro-2*H*-chromen-2-one (2i) [6]: (using general procedure, 110 mg of **2i** was obtained from 155 mg (1 mmol of **1i**) 62% yield; yellow solid, mp 148–150 °C; R_f = 0.4 (EtOAc/hexanes = 2:8); ^{1}H NMR (300 MHz, CDCl$_3$) δ: 7.6 (d, 1H, *J* = 9.7 Hz); 7.5 (d, 1H, *J* = 2.6 Hz); 7.4 (d, 1H, *J* = 9.7 Hz); 7.29 (d, 1H, *J* = 9.59 Hz); 6.47 (d, 1H, *J* = 9.59 Hz); ^{13}C NMR (75 MHz, DMSO-d_6) δ: 159.4, 152.1, 142.9, 131.4, 128.2, 127.4, 120.6, 118.2, 117.3; IR (KBr) ν: 2899, 1726, 1216, 768; ESIMS m/z [M + H]$^+$: 181.0; HRMS (ESI) *m*/*z* calcd for $C_9H_5ClO_2$ [M + H]$^+$: 181.0012, found:181.0055.

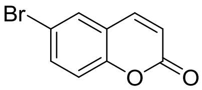

6-Bromo-2*H*-chromen-2-one (2j) [7]: (using general procedure, 138 mg of **2j** was obtained from 199 mg (1 mmol of **1j**) 62% yield; white solid, mp 168–170 °C; R_f = 0.5 (EtOAc/hexanes = 2:8); ^{1}H NMR (300 MHz, CDCl$_3$) δ: 7.65–7.60 (m, 3H); 7.2 (d, 1H, *J* = 9.4 Hz); 6.8 (d, 1H, *J* = 9.4 Hz); ^{13}C NMR (50 MHz, CDCl$_3$) δ: 159.9, 152.9, 142.1, 134.6, 130.2, 120.3, 118.6, 117.8, 117.0; ESIMS *m*/*z* [M + H]$^+$: 225.1.

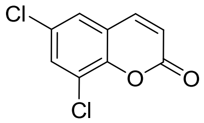

6,8-Dichloro-2*H*-chromen-2-one (2k) [8]: (using general procedure, 127 mg of **2k** was obtained from 189 mg (1 mmol of **1k**) 60% yield; light brown solid, mp 148–150 °C; R_f = 0.4 (EtOAc/hexanes = 2:8); ^{1}H NMR (300 MHz, DMSO-d_6) δ: 8.0 (d, 1H, *J* = 9.6 Hz); 7.9 (d, 1H, *J* = 2.3 Hz); 7.8 (d, 1H, *J* = 2.3 Hz); 6.6 (d, 1H, *J* = 9.6 Hz); ^{13}C NMR (75 MHz, DMSO-d_6) δ: 158.3, 147.8, 142.7, 130.9, 128.1, 126.66, 121.0, 120.8, 118.0; IR(KBr) ν: 2260, 1738, 1646, 1216, 1027, 997, 761; ESIMS *m*/*z* $[M + H]^+$: 214.

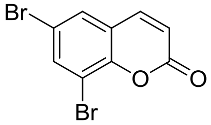

6,8-Dibromo-2*H*-chromen-2-one (2l) [9]: (using general procedure, 195 mg of **2l** was obtained from 277 mg (1 mmol of **1l**) 65% yield; light yellow solid, mp 164–165 °C; R_f = 0.5 (EtOAc/hexanes = 2:8); ^{1}H NMR (300 MHz, DMSO-d_6) δ: 8.3(d, 1H, *J* = 2.06 Hz); 8.03 (d, 1H, *J* = 9.6 Hz); 8.01 (d, 1H, *J* = 2.06 Hz); 6.6 (d, 1H, *J* = 9.6 Hz); ^{13}C NMR (50 MHz, DMSO-d_6) δ: 158.6, 149.4, 142.8, 136.3, 130.2, 121.5, 117.9, 116.1, 110.3; IR (KBr) ν: 3019, 1742, 1639, 1361, 1217, 764.

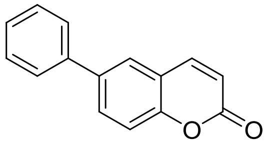

6-Phenyl-2*H*-chromen-2-one (2m) [10]: (using general procedure, 168 mg of **2m** was obtained from 198 mg (1 mmol of **1m**) 76% yield; white solid, mp 112–114 °C; R_f = 0.4 (EtOAc/hexanes = 2:8); ^{1}H NMR (300 MHz, $CDCl_3$) δ: 7.7 (m, 2H); 7.6 (s, 1H); 7.5 (d, 2H, *J* = 7.2 Hz); 7.4 (t, 2H, *J* = 7.5 Hz); 7.4 (d, 2H, *J* = 9.5 Hz); 6.4 (d, 1H, *J* = 9.5 Hz); ^{13}C NMR (50 MHz, $CDCl_3$) δ: 160.7, 153.3, 143.5, 139.3, 137.7, 130.7, 129.0, 127.8, 127.0, 126.0, 119.0, 117.2, 116.9.

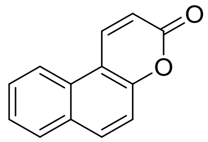

3*H*-Benzo[*f*]chromen-3-one (2n) [11]: (using general procedure, 127 mg of **2n** was obtained from 172 mg (1 mmol) of **1n**) 65% yield; white solid, mp 110–111 °C; R_f = 0.7 (EtOAc/hexanes = 2:8); ^{1}H NMR (300 MHz, $CDCl_3$) δ: 7.8 (m, 2H); 7.7 (d, 1H, *J* = 7.8 Hz); 7.5 (d, 1H, *J* = 1.3 Hz); 7.4 (m, 4H); ^{13}C NMR (75 MHz, $CDCl_3$) δ: 151.6, 134.1, 130.3, 129.8, 127.7, 127.3, 126.6, 124.9, 119.6, 113.2, 111.1.

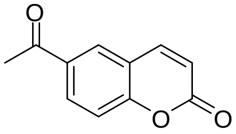

6-Acetyl-2*H*-chromen-2-one (2o): (using general procedure, 157 mg of **2o** was obtained from 164 mg (1 mmol) of **1o**) 84% yield; Colorless oil; R_f = 0.4 (EtOAc/hexanes = 2:8); ^{1}H NMR (300 MHz, $CDCl_3$) δ: 8.1(d, 2H, *J* = 6 Hz); 7.8 (d, 1H, *J* = 9.0 Hz); 7.4 (d, 1H, *J* = 9 Hz); 6.4 (d, 1H, *J* = 9.0 Hz); 2.6 (s, 3H).

References:

1. Nitin, T. P.; Vivek, S. R. *J. Org. Chem*. **2010**, *75*, 6961–6964.

2. Dittmer, D. C.; Li, Q.; Avilov, D. V. *J. Org. Chem*. **2005**, *70*, 4682–4686.

3. Anwar, H. F.; Skattebol, A.; Hansen; T. V. *Tetrahedron Lett.* **2005**, *46*, 5285– 5287.

4. Juzo, O.; Tsuzio, K. *Tetrahedron* **2006**, *62*, 6918– 6925.

5. Rajeev, S. M.; Findlay, A. D.; Bissember, A. C.; Banwell, M. G. *J. Org. Chem.* **2009**, *74*, 8901–8903.

6. Zeitler, K.; Rose, A. C. *J. Org. Chem.* **2009,** *74*, 1759–1762.

7. Dubuffet, T.; Luotz, A.; Lavielle, G. *Synth. Commun* **1999**, *29*, 929 – 936.

8. Nagarajan, S. R. et al. *Bioorg. Med. Chem.* **2007**, *15*, 3783–3800.

9. Schmidt, B.; Krehl, S. *Chem.Commun*, **2011**, *47*, 5879–5881.

10. Stercevic, S.; Brozic, P.; Turk, S.; Cesar, J.; Rizner, T. L.;Gobec, S. *J. Med. Chem*. **2011**, *54*, 248–261.

11. Trost, B. M.; Toste, F. D.; Greenman, K. *J. Am. Chem. Soc.* **2003**, *125*, 4518–4526.

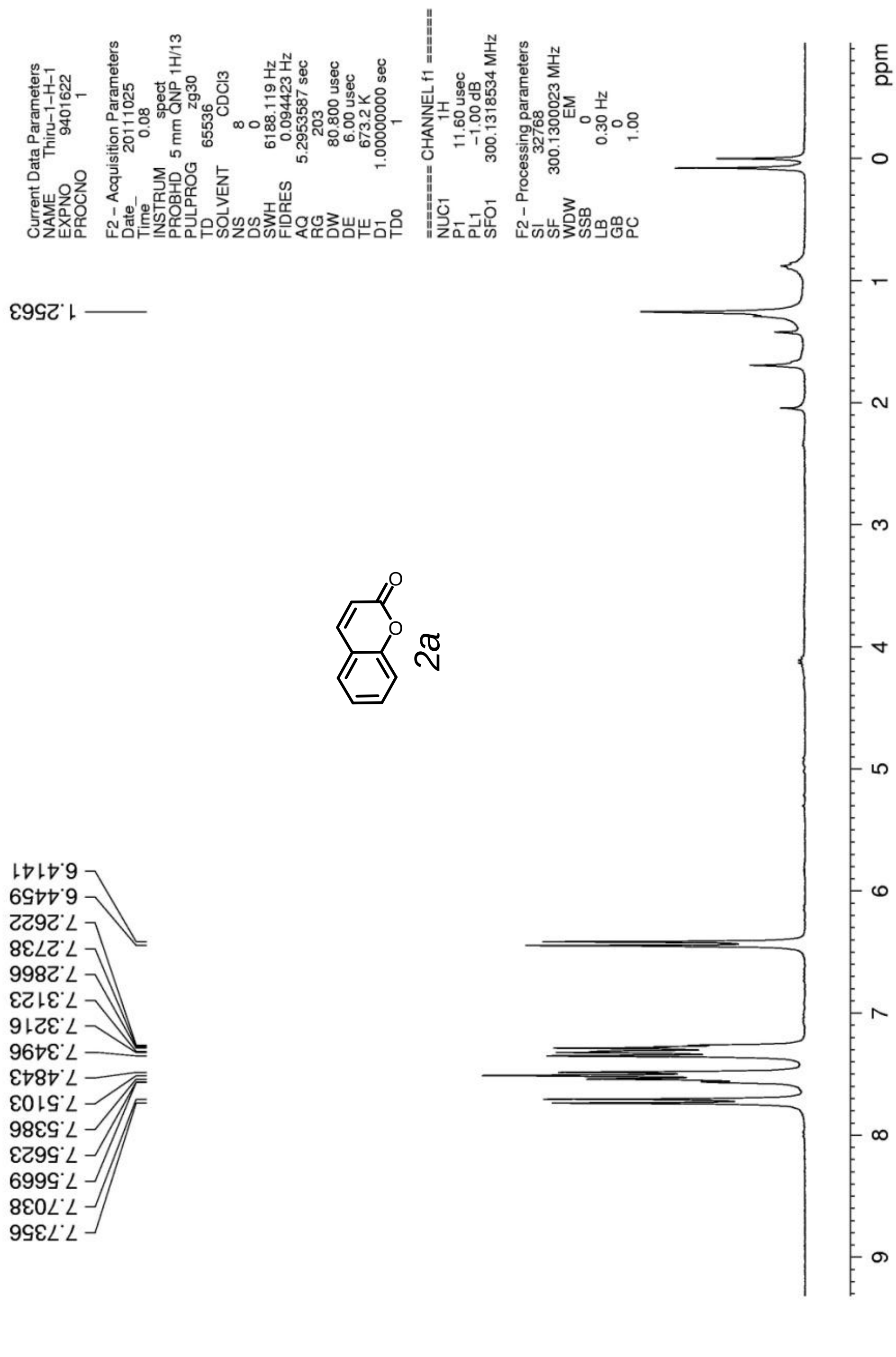

2a
Current Data Parameters
NAME Thiru-1-H-1
EXPNO 9401622
PROCNO 1
F2 – Acquisition Parameters
Date_ 20111025
Time 0.08
INSTRUM spect
PROBHD 5 mm QNP 1H/13
PULPROG zg30
TD 65536
SOLVENT CDCl3
NS 8
DS 0
SWH 6188.119 Hz
FIDRES 0.094423 Hz
AQ 5.2953587 sec
RG 203
DW 80.800 usec
DE 6.00 usec
TE 673.2 K
D1 1.00000000 sec
TD0 1
======== CHANNEL f1 ========
NUC1 1H
P1 11.60 usec
PL1 -1.00 dB
SFO1 300.1318534 MHz
F2 – Processing parameters
SI 32768
SF 300.1300023 MHz
WDW EM
SSB 0
LB 0.30 Hz
GB 0
PC 1.00
1.2563
6.4141
6.4459
7.2622
7.2738
7.2866
7.3123
7.3216
7.3496
7.4843
7.5103
7.5386
7.5623
7.5669
7.7038
7.7356
1.00
2.11
2.01
1.03
ppm

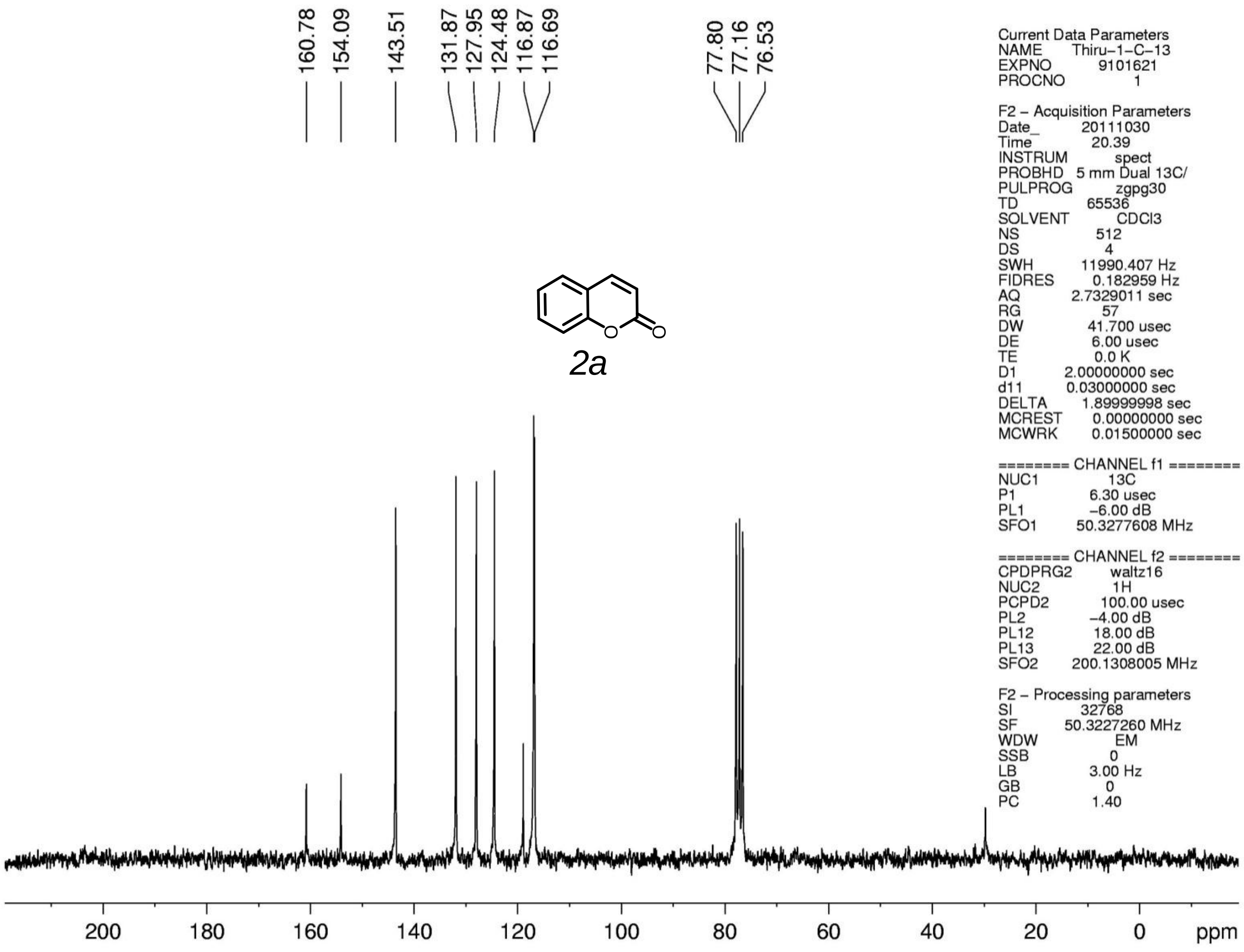
160.78
154.09
143.51
131.87
127.95
124.48
116.87
116.69
77.80
77.16
76.53
2a
Current Data Parameters
NAME Thiru-1-C-13
EXPNO 9101621
PROCNO 1
F2 - Acquisition Parameters
Date_ 20111030
Time 20.39
INSTRUM spect
PROBHD 5 mm Dual 13C/
PULPROG zgpg30
TD 65536
SOLVENT CDCl3
NS 512
DS 4
SWH 11990.407 Hz
FIDRES 0.182959 Hz
AQ 2.7329011 sec
RG 57
DW 41.700 usec
DE 6.00 usec
TE 0.0 K
D1 2.00000000 sec
d11 0.03000000 sec
DELTA 1.89999998 sec
MCREST 0.00000000 sec
MCWRK 0.01500000 sec
======== CHANNEL f1 ========
NUC1 13C
P1 6.30 usec
PL1 -6.00 dB
SFO1 50.3277608 MHz
======== CHANNEL f2 ========
CPDPRG2 waltz16
NUC2 1H
PCPD2 100.00 usec
PL2 -4.00 dB
PL12 18.00 dB
PL13 22.00 dB
SFO2 200.1308005 MHz
F2 - Processing parameters
SI 32768
SF 50.3227260 MHz
WDW EM
SSB 0
LB 3.00 Hz
GB 0
PC 1.40
200
180
160
140
120
100
80
60
40
20
0
ppm

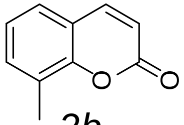

2b

55-124-2

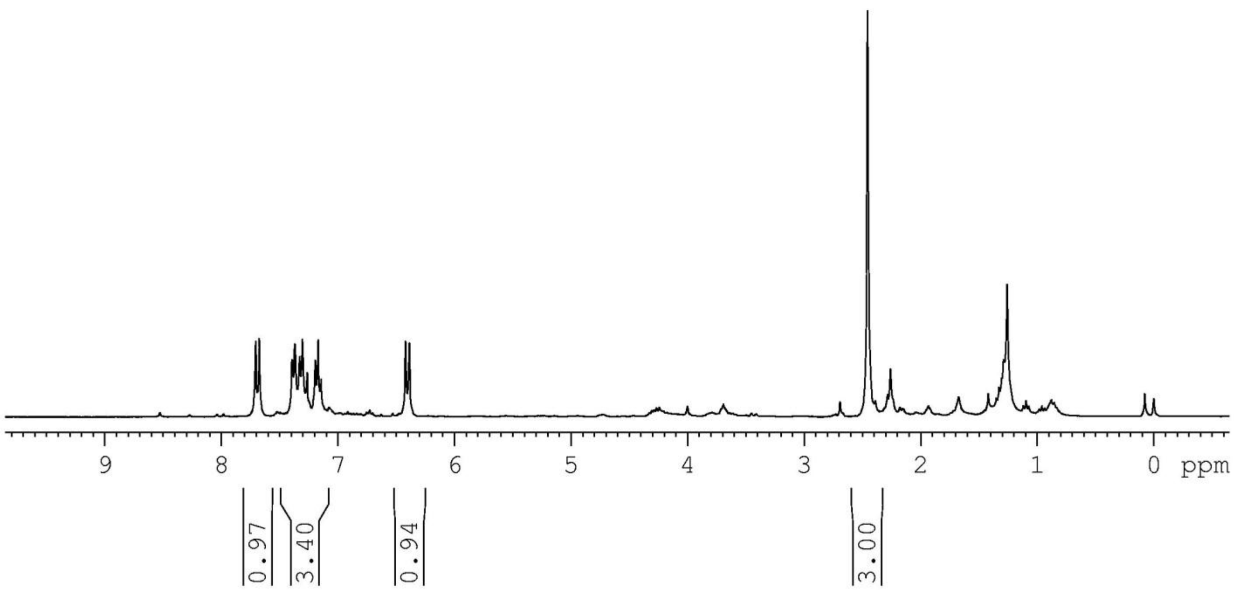


```
Current Data Paramete
NAME      New Folder (
EXPNO          9305512
PROCNO

F2 - Acquisition Para
Date_           201206
Time               16.
INSTRUM            spe
PROBHD   5 mm QNP 1H/
PULPROG             zg
TD                 655
SOLVENT            CDC
NS
DS
SWH             6188.1
FIDRES          0.0944
AQ             5.29535
RG                   1
DW                80.8
DE                  6.
TE                 303
D1            1.000000
TD0

======== CHANNEL f1 =
NUC1
P1                 11.
PL1                -1.
SFO1         300.13185

F2 - Processing param
SI                 327
SF           300.13000
WDW
SSB
```

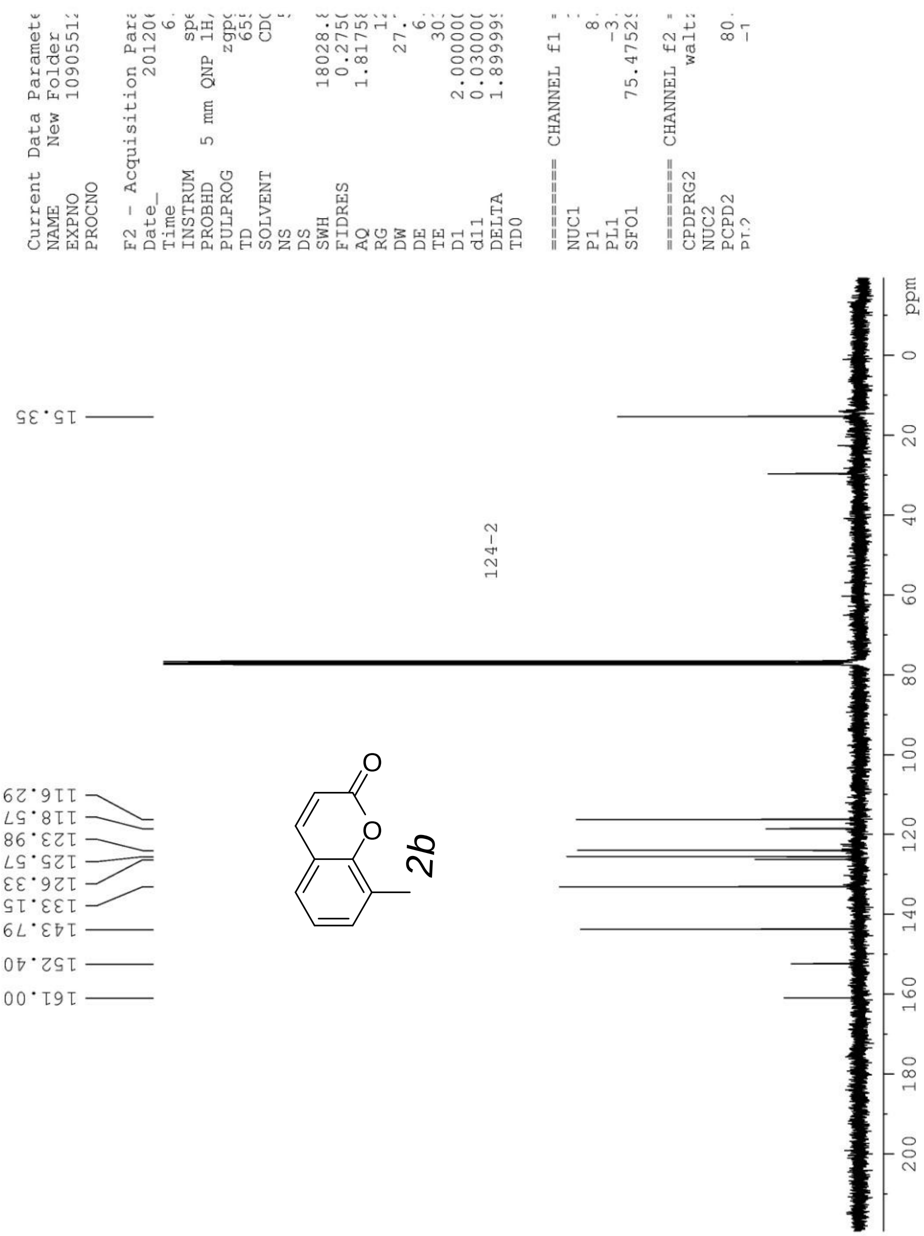
Current Data Paramete
NAME New Folder
EXPNO 109055l
PROCNO
F2 - Acquisition Para
Date_ 20120
Time 6.
INSTRUM spe
PROBHD 5 mm QNP 1H,
PULPROG zgp
TD 65
SOLVENT CD
NS
DS
SWH 18028.
FIDRES 0.275
AQ 1.8175
RG 1
DW 27.
DE 6.
TE 30
D1 2.00000
d11 0.03000
DELTA 1.89999
TD0
======== CHANNEL f1 =
NUC1
P1 8.
PL1 -3.
SFO1 75.4752
======== CHANNEL f2 =
CPDPRG2 walt
NUC2
PCPD2 80.
PL2 -1
15.35
116.29
118.57
123.98
125.57
126.33
133.15
143.79
152.40
161.00
124-2
O
O
2b
ppm
0
20
40
60
80
100
120
140
160
180
200

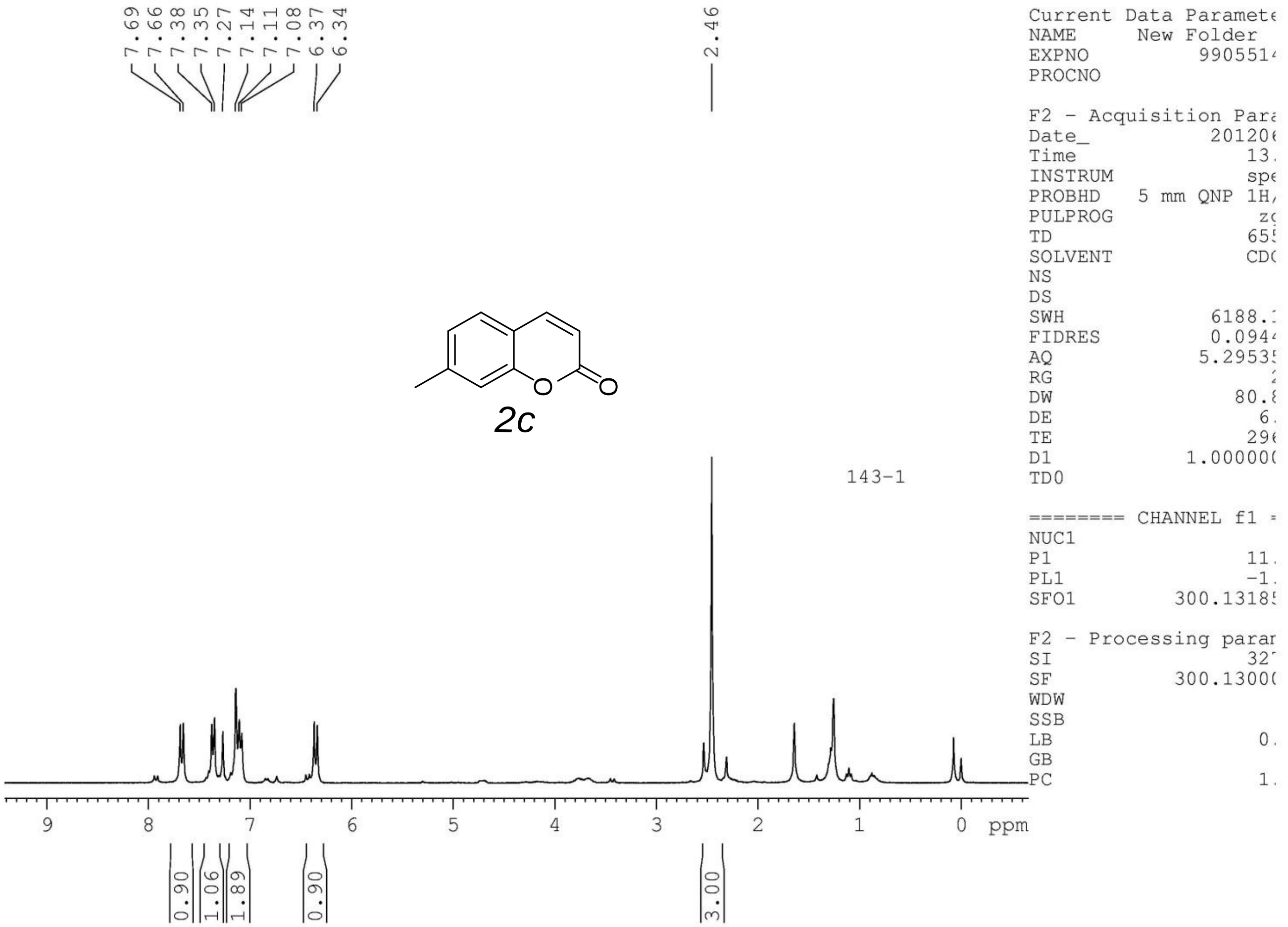
7.69
7.66
7.38
7.35
7.27
7.14
7.11
7.08
6.37
6.34
2.46
2c
143-1
Current Data Paramete
NAME New Folder
EXPNO 990551
PROCNO
F2 - Acquisition Para
Date_ 20120
Time 13.
INSTRUM spe
PROBHD 5 mm QNP 1H,
PULPROG z
TD 65
SOLVENT CD
NS
DS
SWH 6188.
FIDRES 0.094
AQ 5.2953
RG
DW 80.
DE 6.
TE 29
D1 1.00000
TD0
======== CHANNEL f1
NUC1
P1 11.
PL1 -1.
SFO1 300.1318
F2 - Processing para
SI 32
SF 300.1300
WDW
SSB
LB 0.
GB
PC 1.
9
8
7
6
5
4
3
2
1
0
ppm
0.90
1.06
1.89
0.90
3.00

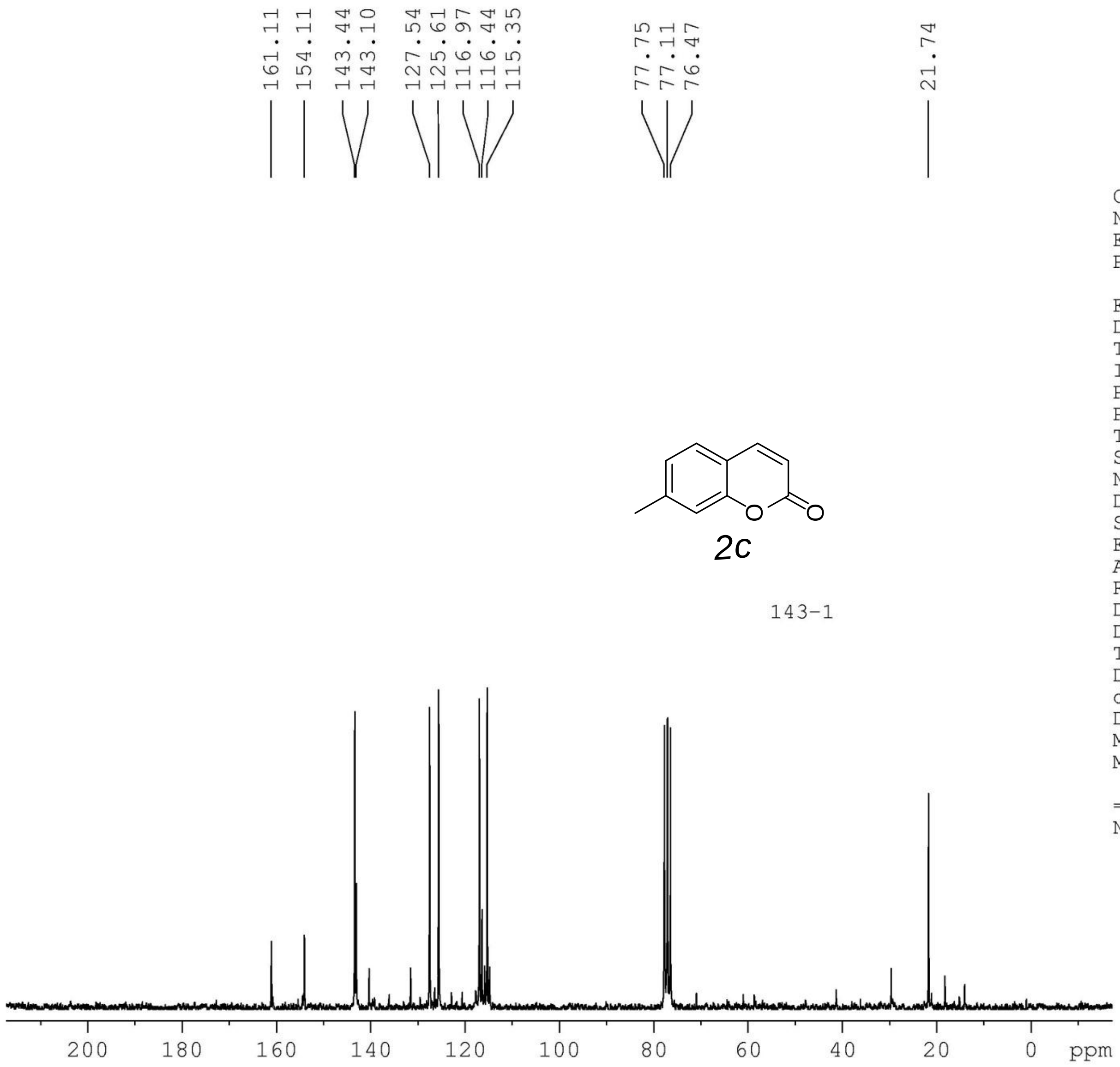

2c

143-1

Current Data Paramete	
NAME	Jul03-2012-D
EXPNO	9
PROCNO	
F2 - Acquisition Para	
Date_	201301
Time	11.
INSTRUM	spe
PROBHD	5 mm Dual 13
PULPROG	zgpg
TD	655
SOLVENT	CDC
NS	8
DS	
SWH	11990.4
FIDRES	0.1829
AQ	2.73290
RG	50
DW	41.7
DE	6.
TE	0
D1	2.000000
d11	0.030000
DELTA	1.899999
MCREST	0.000000
MCWRK	0.015000
======== CHANNEL f1 =	
NUC1	1

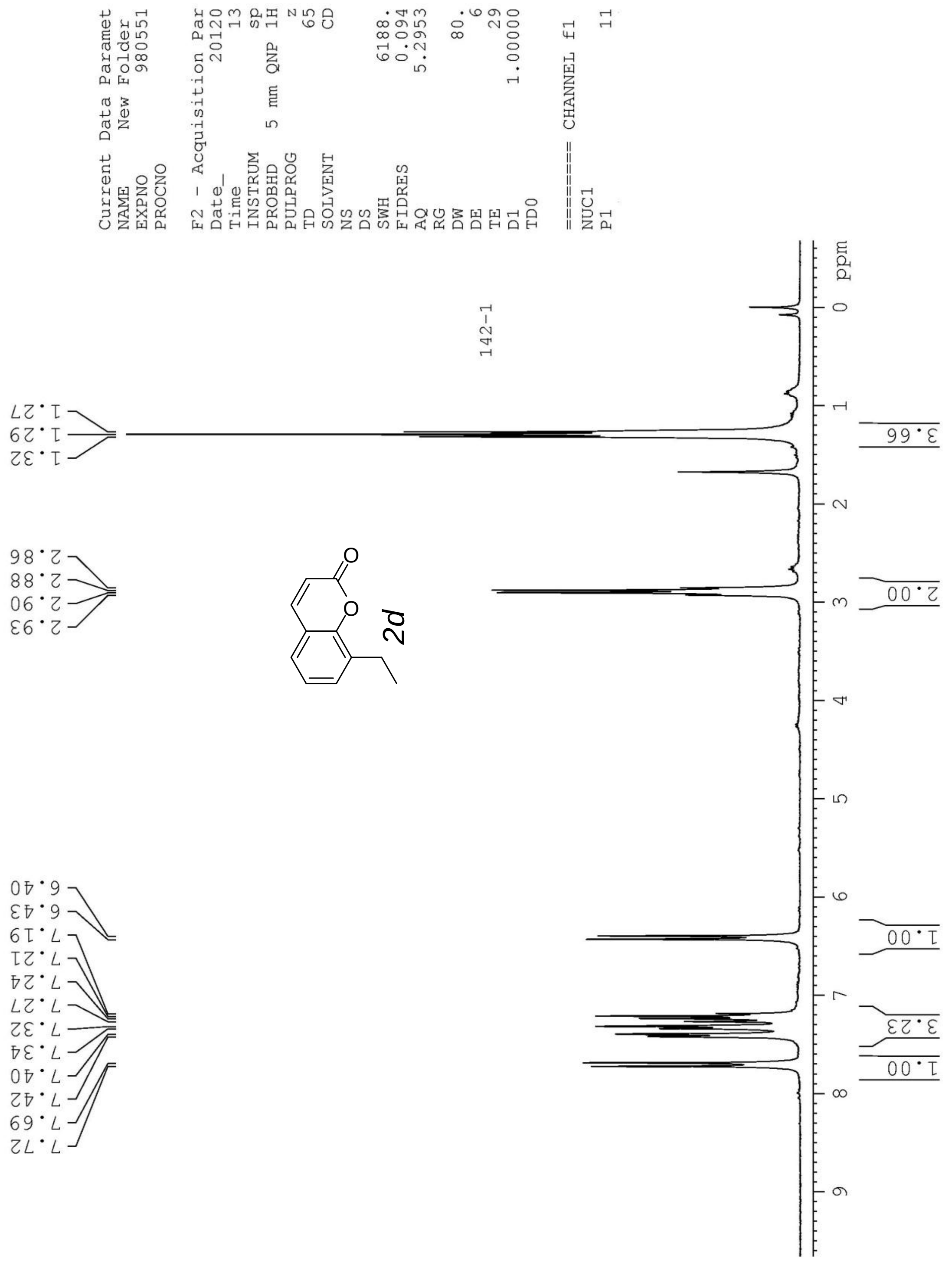
Current Data Paramet
NAME New Folder
EXPNO 980551
PROCNO
F2 - Acquisition Par
Date_ 20120
Time 13
INSTRUM sp
PROBHD 5 mm QNP 1H
PULPROG z
TD 65
SOLVENT CD
NS
DS
SWH 6188.
FIDRES 0.094
AQ 5.2953
RG
DW 80.
DE 6
TE 29
D1 1.00000
TD0
======== CHANNEL f1
NUC1
P1 11
142-1
2d
1.27
1.29
1.32
2.86
2.88
2.90
2.93
6.40
6.43
7.19
7.21
7.24
7.27
7.32
7.34
7.40
7.42
7.69
7.72
3.66
2.00
1.00
3.23
1.00
ppm

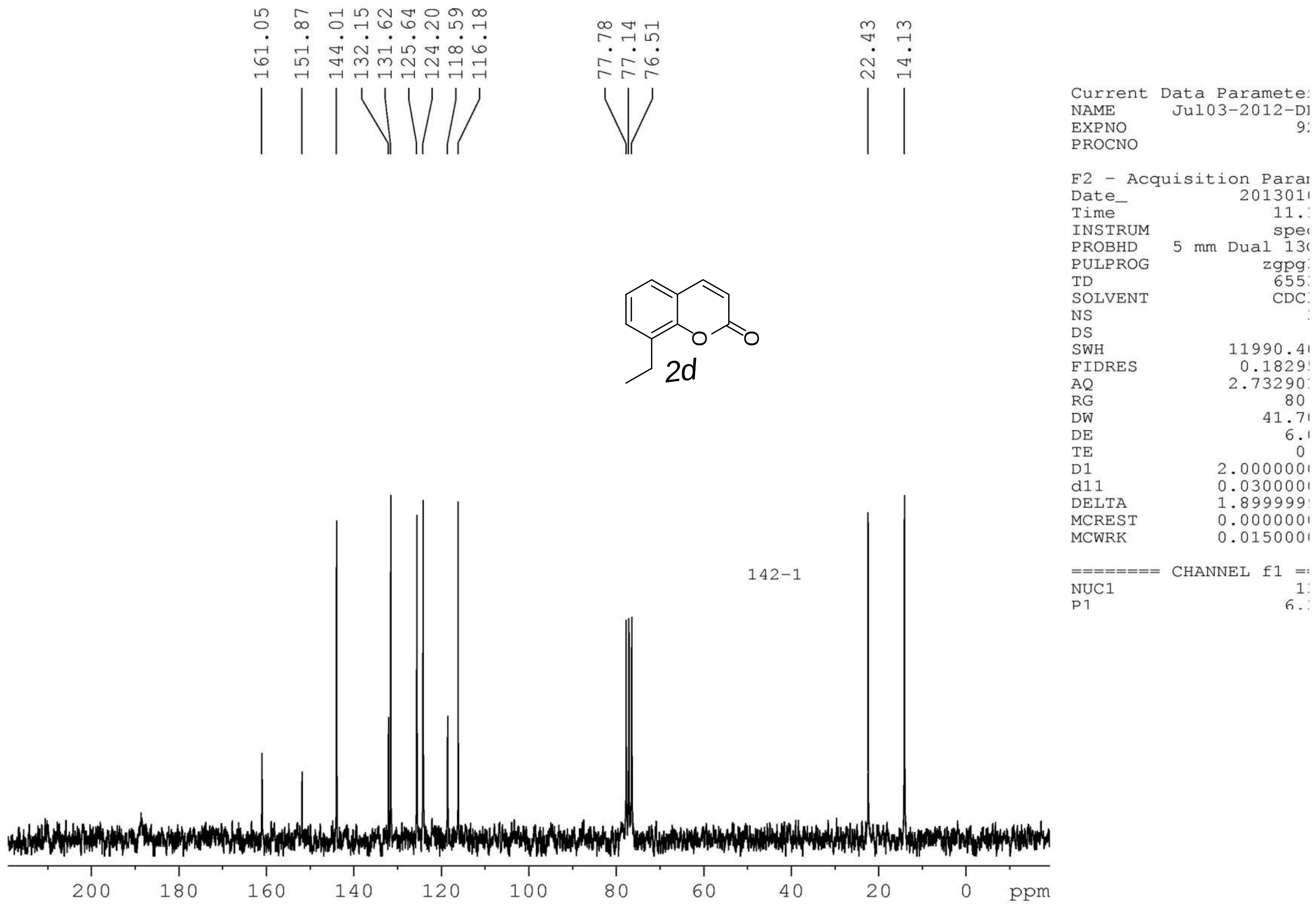
161.05
151.87
144.01
132.15
131.62
125.64
124.20
118.59
116.18
77.78
77.14
76.51
22.43
14.13
2d
142-1
Current Data Paramete
NAME Jul03-2012-D
EXPNO 9
PROCNO
F2 - Acquisition Para
Date_ 201301
Time 11.
INSTRUM spe
PROBHD 5 mm Dual 13
PULPROG zgpg
TD 655
SOLVENT CDC
NS
DS
SWH 11990.4
FIDRES 0.1829
AQ 2.73290
RG 80
DW 41.7
DE 6.
TE 0
D1 2.000000
d11 0.030000
DELTA 1.899999
MCREST 0.000000
MCWRK 0.015000
======== CHANNEL f1 =
NUC1 1
P1 6.
200
180
160
140
120
100
80
60
40
20
0
ppm

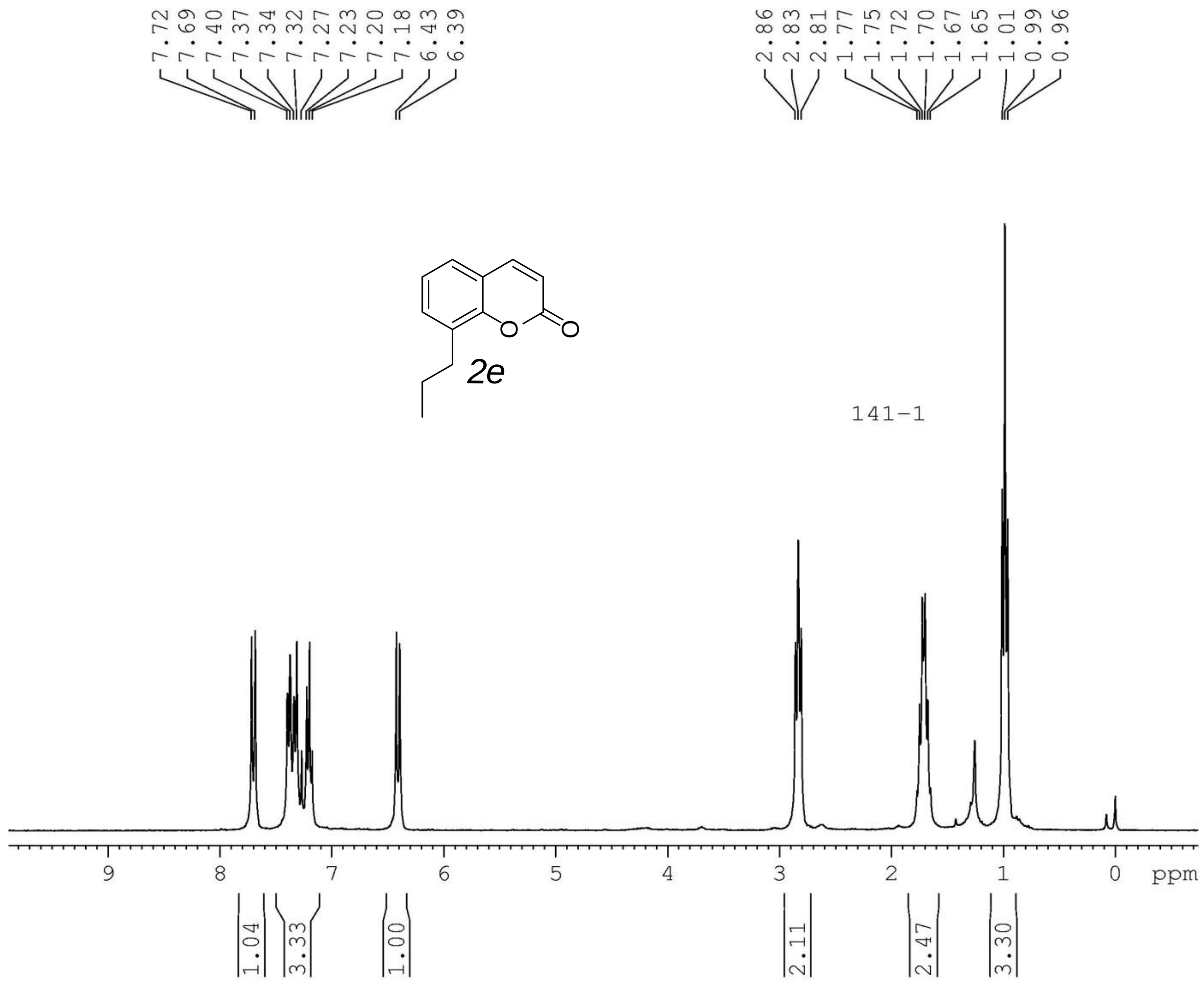
7.72
7.69
7.40
7.37
7.34
7.32
7.27
7.23
7.20
7.18
6.43
6.39
2.86
2.83
2.81
1.77
1.75
1.72
1.70
1.67
1.65
1.01
0.99
0.96
2e
141-1
9
8
7
6
5
4
3
2
1
0
ppm
1.04
3.33
1.00
2.11
2.47
3.30

Current Data
NAME New
EXPNO
PROCNO
F2 - Acquisi
Date_
Time
INSTRUM
PROBHD 5 m
PULPROG
TD
SOLVENT
NS
DS
SWH
FIDRES
AQ
RG
DW
DE
TE
D1
TD0
======== CHA

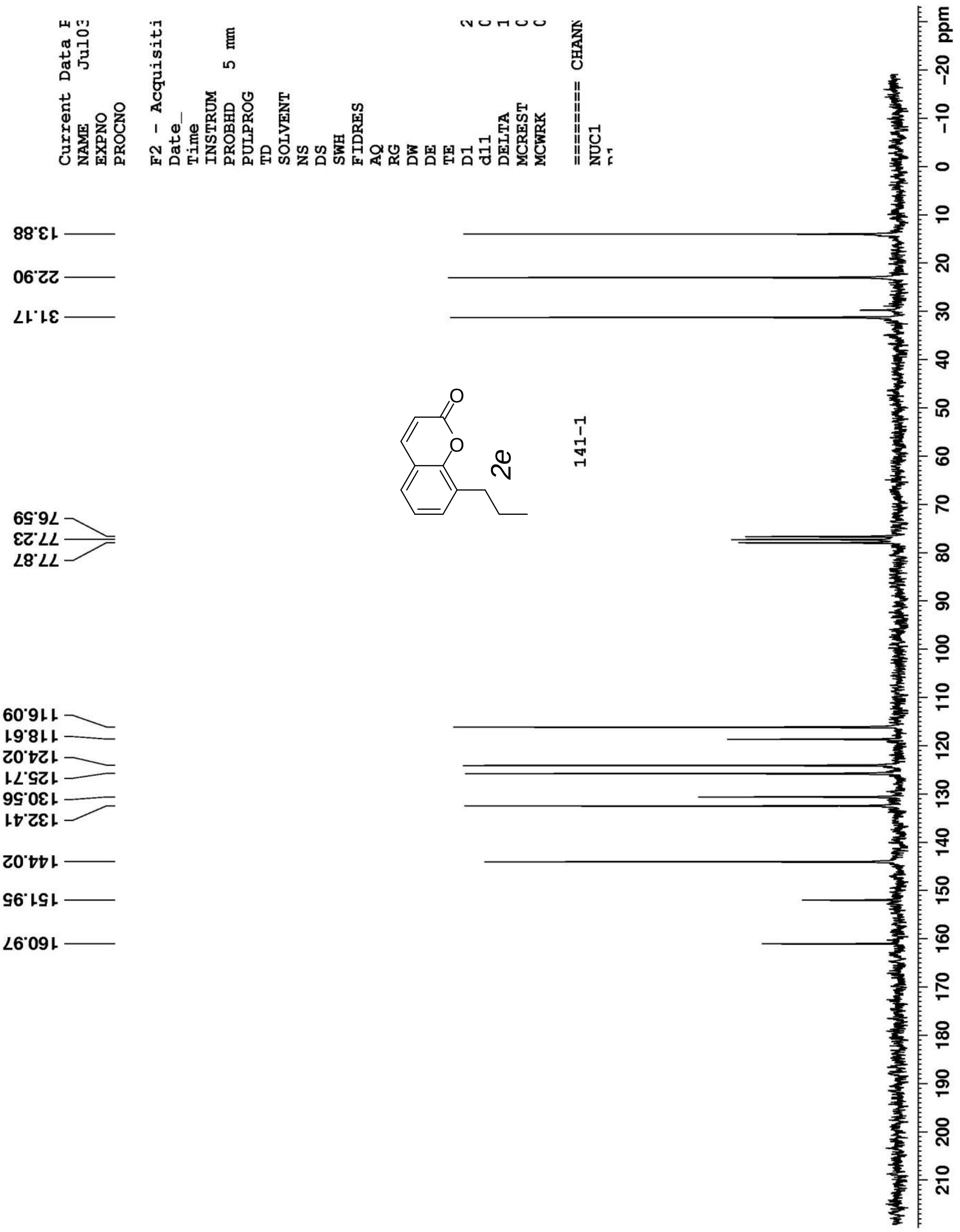
Current Data P
NAME Jul03
EXPNO
PROCNO
F2 - Acquisiti
Date_
Time
INSTRUM
PROBHD 5 mm
PULPROG
TD
SOLVENT
NS
DS
SWH
FIDRES
AQ
RG
DW
DE
TE
D1
d11
DELTA
MCREST
MCWRK
======== CHANN
NUC1
160.97
151.95
144.02
132.41
130.56
125.71
124.02
118.61
116.09
77.87
77.23
76.59
31.17
22.90
13.88
2e
141-1
O
210 200 190 180 170 160 150 140 130 120 110 100 90 80 70 60 50 40 30 20 10 0 -10 -20 ppm

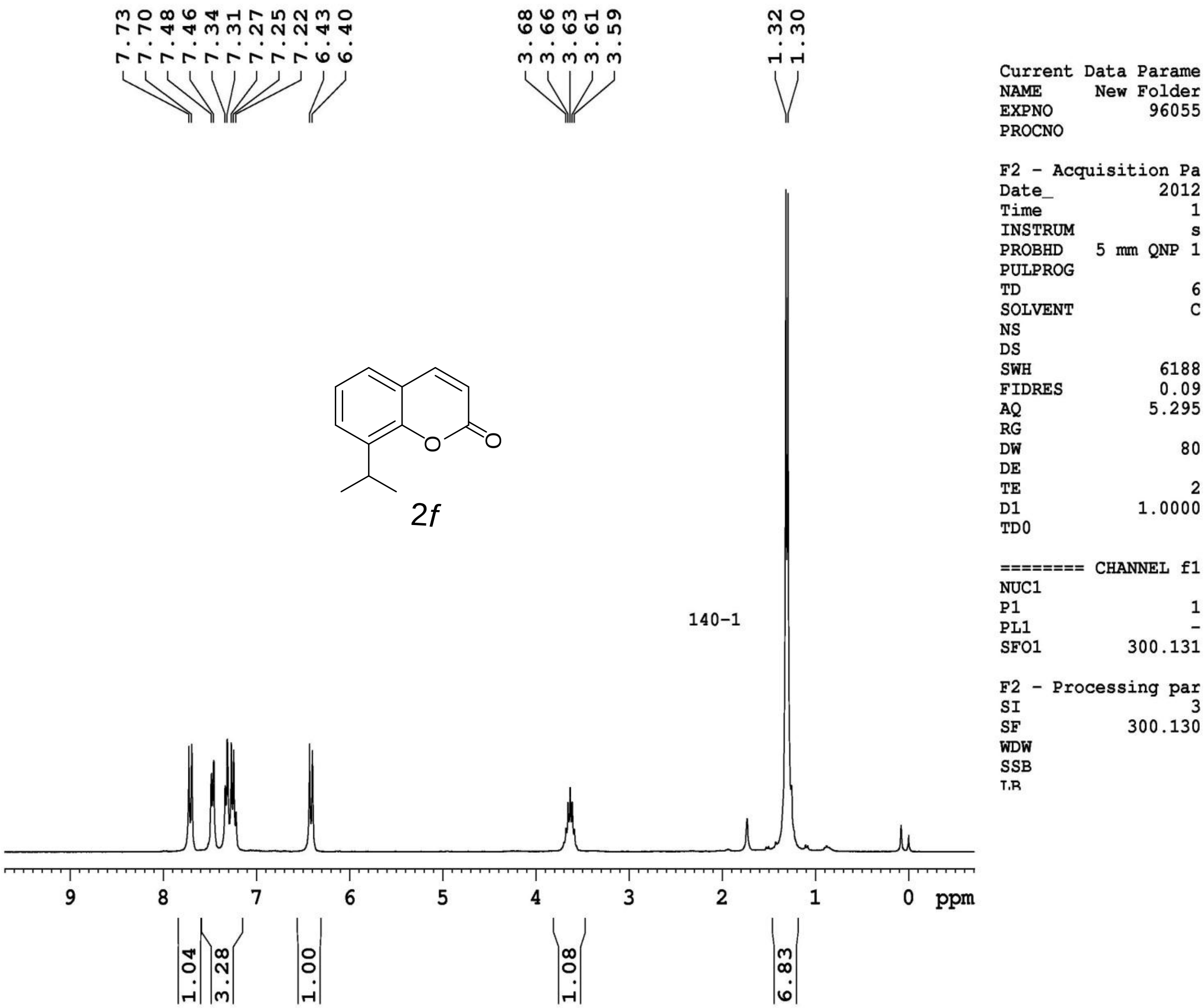
7.73
7.70
7.48
7.46
7.34
7.31
7.27
7.25
7.22
6.43
6.40
3.68
3.66
3.63
3.61
3.59
1.32
1.30
2f
140-1
9 8 7 6 5 4 3 2 1 0 ppm
1.04
3.28
1.00
1.08
6.83
Current Data Parame
NAME New Folder
EXPNO 96055
PROCNO
F2 - Acquisition Pa
Date_ 2012
Time 1
INSTRUM s
PROBHD 5 mm QNP 1
PULPROG
TD 6
SOLVENT C
NS
DS
SWH 6188
FIDRES 0.09
AQ 5.295
RG
DW 80
DE
TE 2
D1 1.0000
TD0
======== CHANNEL f1
NUC1
P1 1
PL1 -
SFO1 300.131
F2 - Processing par
SI 3
SF 300.130
WDW
SSB

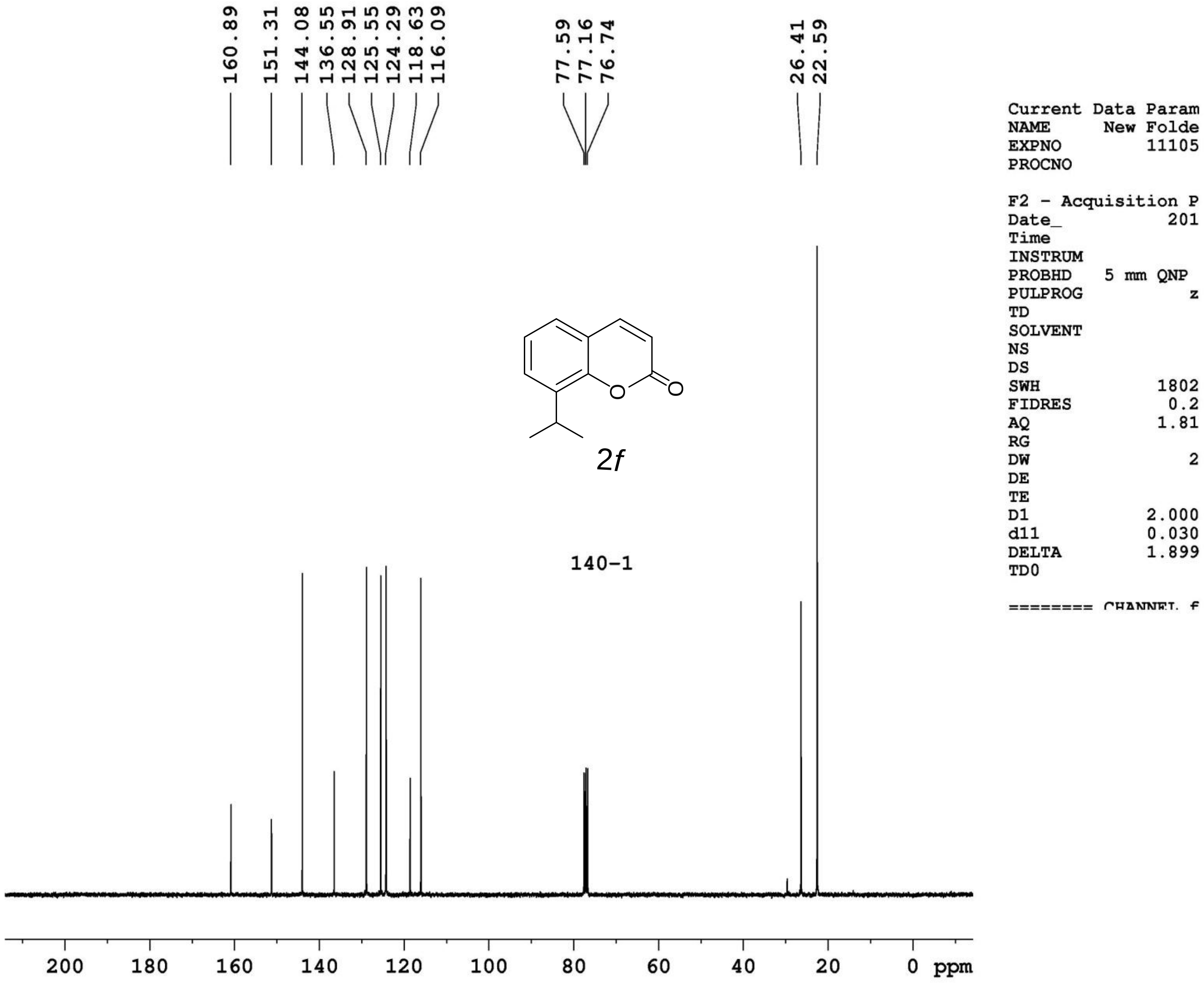
160.89
151.31
144.08
136.55
128.91
125.55
124.29
118.63
116.09
77.59
77.16
76.74
26.41
22.59
2f
140-1
Current Data Param
NAME New Folde
EXPNO 11105
PROCNO
F2 - Acquisition P
Date_ 201
Time
INSTRUM
PROBHD 5 mm QNP
PULPROG z
TD
SOLVENT
NS
DS
SWH 1802
FIDRES 0.2
AQ 1.81
RG
DW 2
DE
TE
D1 2.000
d11 0.030
DELTA 1.899
TD0
200 180 160 140 120 100 80 60 40 20 0 ppm

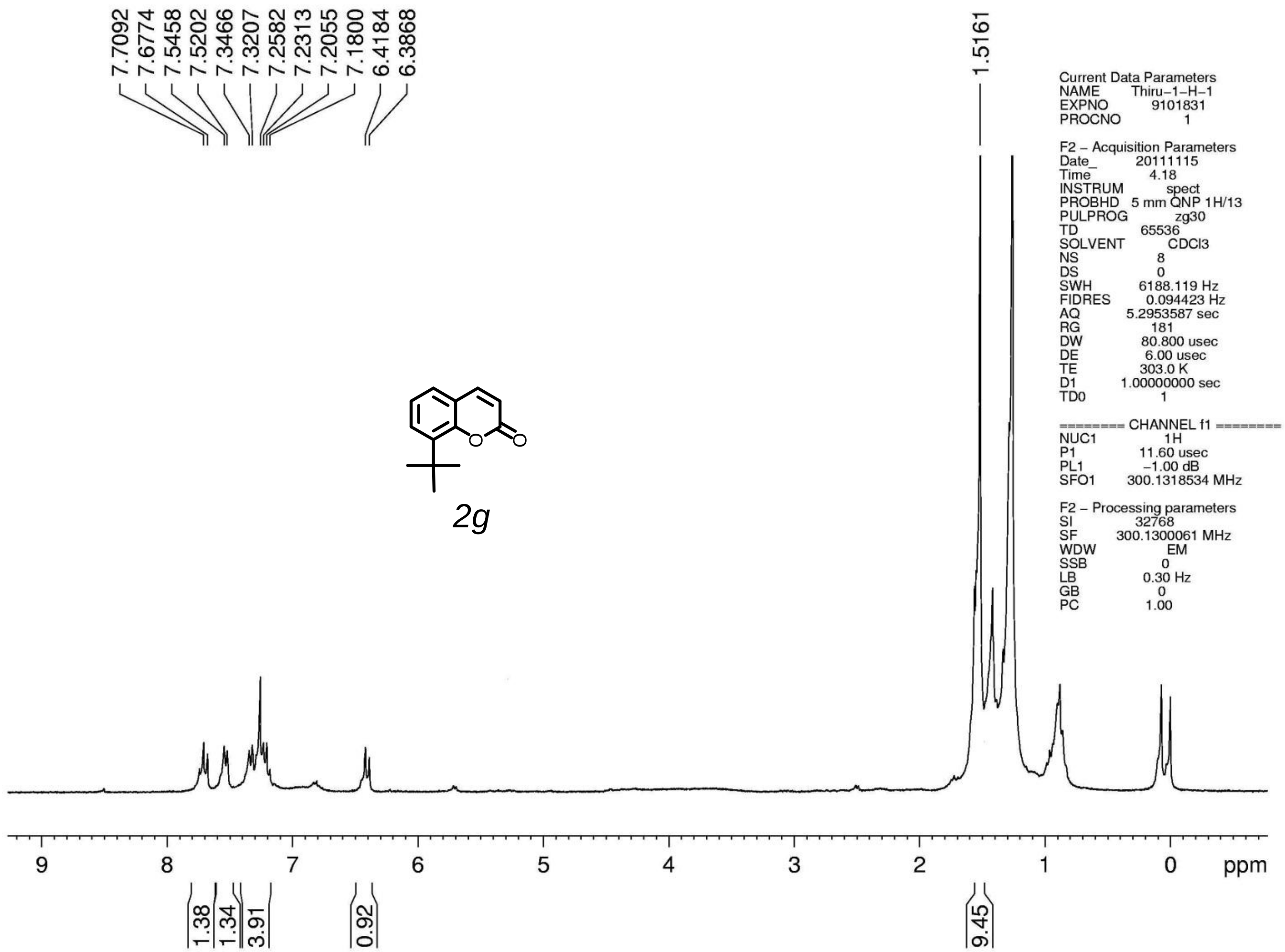
7.7092
7.6774
7.5458
7.5202
7.3466
7.3207
7.2582
7.2313
7.2055
7.1800
6.4184
6.3868
1.5161
2g
Current Data Parameters
NAME Thiru-1-H-1
EXPNO 9101831
PROCNO 1
F2 - Acquisition Parameters
Date_ 20111115
Time 4.18
INSTRUM spect
PROBHD 5 mm QNP 1H/13
PULPROG zg30
TD 65536
SOLVENT CDCl3
NS 8
DS 0
SWH 6188.119 Hz
FIDRES 0.094423 Hz
AQ 5.2953587 sec
RG 181
DW 80.800 usec
DE 6.00 usec
TE 303.0 K
D1 1.00000000 sec
TD0 1
======== CHANNEL f1 ========
NUC1 1H
P1 11.60 usec
PL1 -1.00 dB
SFO1 300.1318534 MHz
F2 - Processing parameters
SI 32768
SF 300.1300061 MHz
WDW EM
SSB 0
LB 0.30 Hz
GB 0
PC 1.00
9
8
7
6
5
4
3
2
1
0
ppm
1.38
1.34
3.91
0.92
9.45

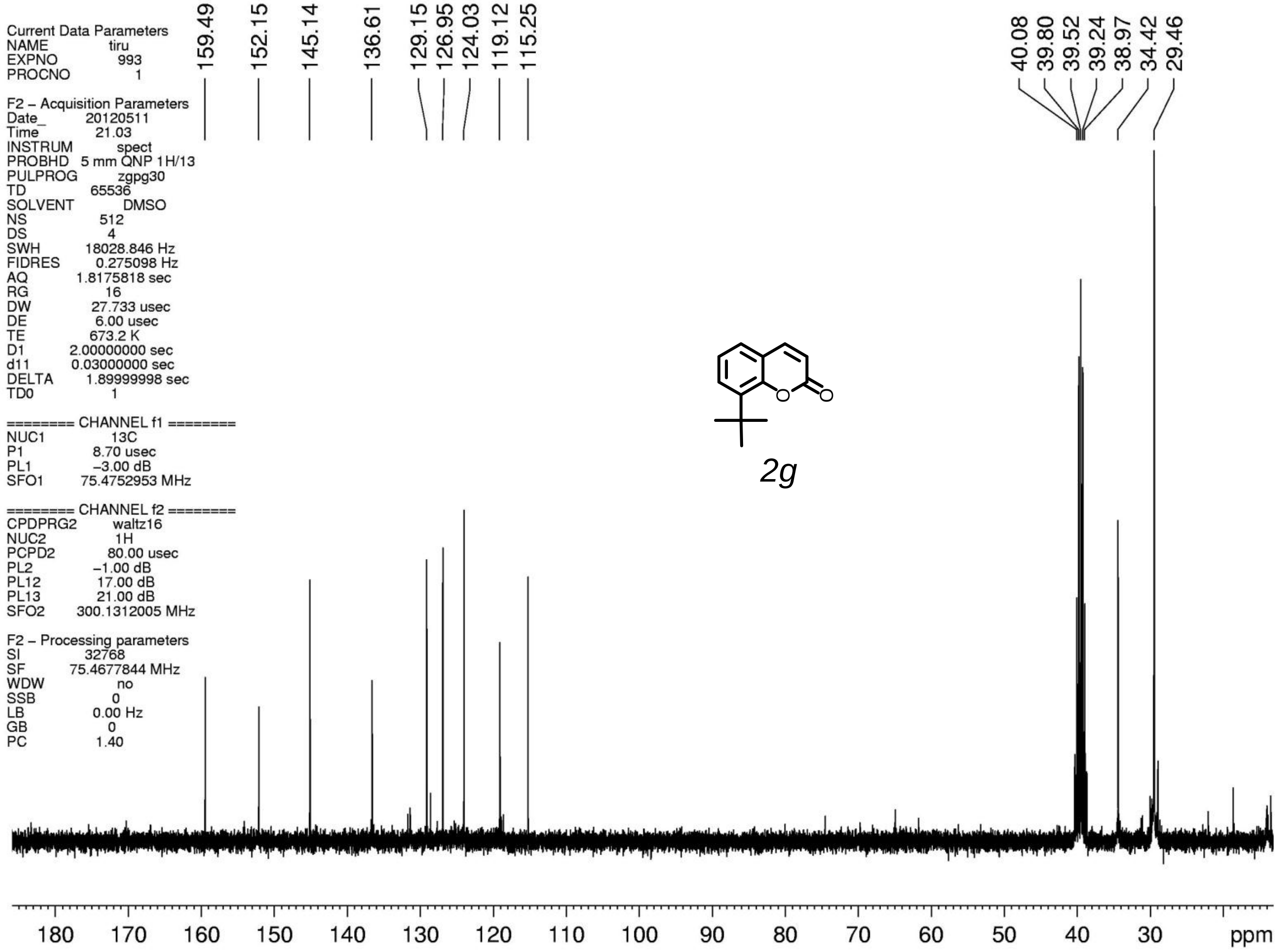
Current Data Parameters
NAME tiru
EXPNO 993
PROCNO 1
F2 – Acquisition Parameters
Date_ 20120511
Time 21.03
INSTRUM spect
PROBHD 5 mm QNP 1H/13
PULPROG zgpg30
TD 65536
SOLVENT DMSO
NS 512
DS 4
SWH 18028.846 Hz
FIDRES 0.275098 Hz
AQ 1.8175818 sec
RG 16
DW 27.733 usec
DE 6.00 usec
TE 673.2 K
D1 2.00000000 sec
d11 0.03000000 sec
DELTA 1.89999998 sec
TD0 1
======== CHANNEL f1 ========
NUC1 13C
P1 8.70 usec
PL1 -3.00 dB
SFO1 75.4752953 MHz
======== CHANNEL f2 ========
CPDPRG2 waltz16
NUC2 1H
PCPD2 80.00 usec
PL2 -1.00 dB
PL12 17.00 dB
PL13 21.00 dB
SFO2 300.1312005 MHz
F2 – Processing parameters
SI 32768
SF 75.4677844 MHz
WDW no
SSB 0
LB 0.00 Hz
GB 0
PC 1.40
159.49
152.15
145.14
136.61
129.15
126.95
124.03
119.12
115.25
40.08
39.80
39.52
39.24
38.97
34.42
29.46
O
O
2g
180 170 160 150 140 130 120 110 100 90 80 70 60 50 40 30 ppm

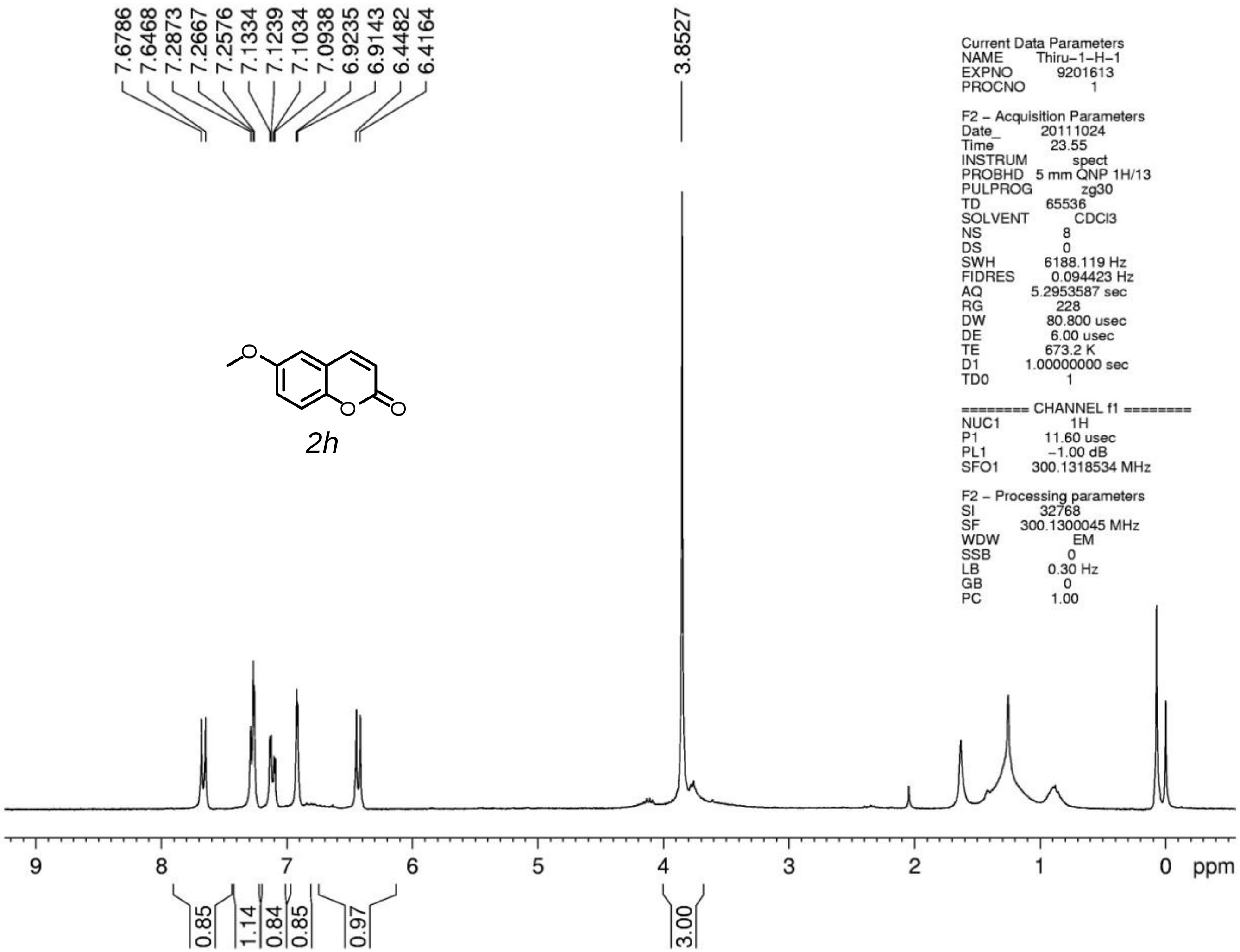
7.6786
7.6468
7.2873
7.2667
7.2576
7.1334
7.1239
7.1034
7.0938
6.9235
6.9143
6.4482
6.4164
3.8527
2h
Current Data Parameters
NAME Thiru-1-H-1
EXPNO 9201613
PROCNO 1
F2 - Acquisition Parameters
Date_ 20111024
Time 23.55
INSTRUM spect
PROBHD 5 mm QNP 1H/13
PULPROG zg30
TD 65536
SOLVENT CDCl3
NS 8
DS 0
SWH 6188.119 Hz
FIDRES 0.094423 Hz
AQ 5.2953587 sec
RG 228
DW 80.800 usec
DE 6.00 usec
TE 673.2 K
D1 1.00000000 sec
TD0 1
======== CHANNEL f1 ========
NUC1 1H
P1 11.60 usec
PL1 -1.00 dB
SFO1 300.1318534 MHz
F2 - Processing parameters
SI 32768
SF 300.1300045 MHz
WDW EM
SSB 0
LB 0.30 Hz
GB 0
PC 1.00
9
8
7
6
5
4
3
2
1
0
ppm
0.85
1.14
0.84
0.85
0.97
3.00

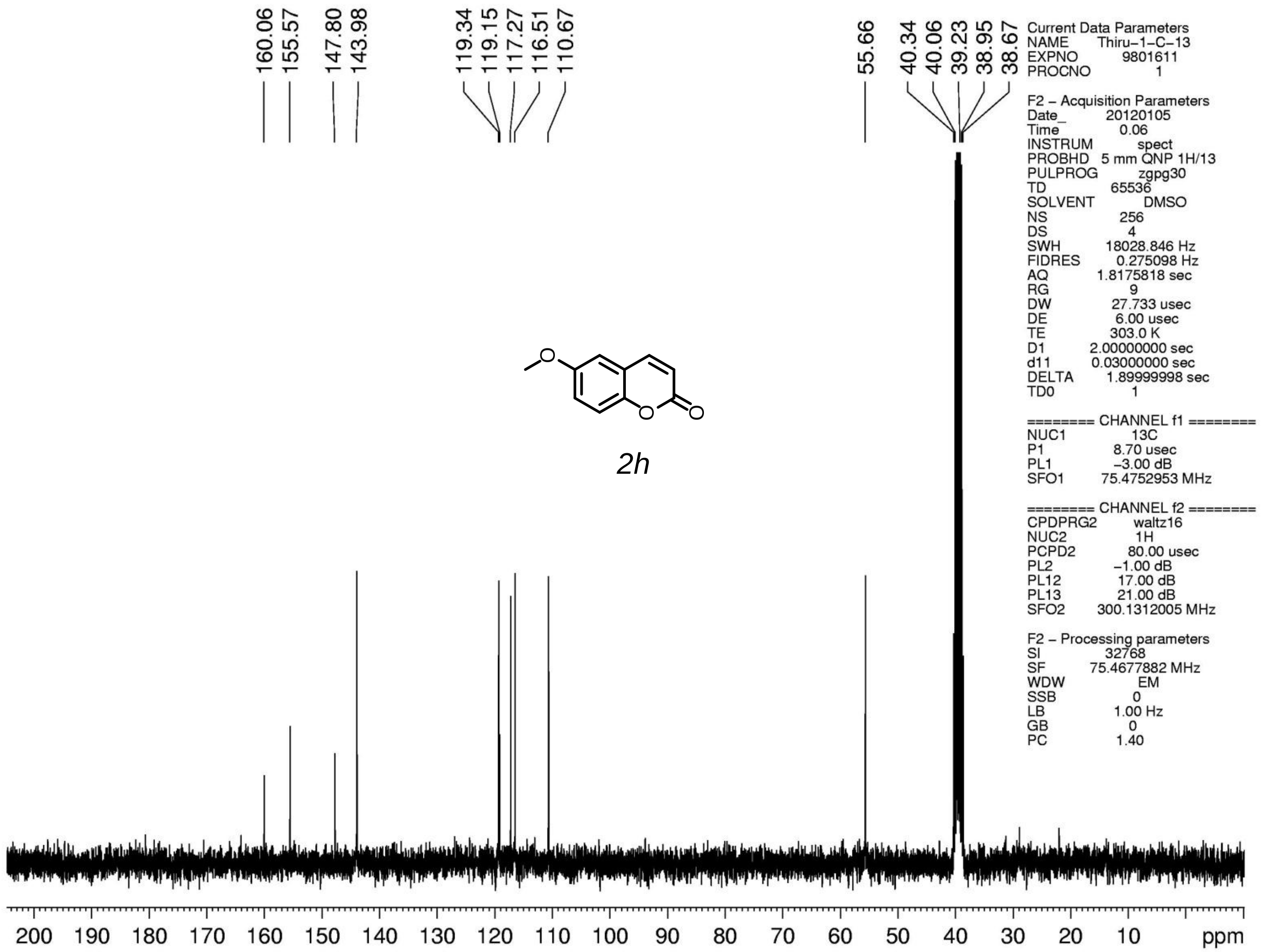

160.06
155.57
147.80
143.98
119.34
119.15
117.27
116.51
110.67
55.66
40.34
40.06
39.23
38.95
38.67
Current Data Parameters
NAME Thiru-1-C-13
EXPNO 9801611
PROCNO 1
F2 - Acquisition Parameters
Date_ 20120105
Time 0.06
INSTRUM spect
PROBHD 5 mm QNP 1H/13
PULPROG zgpg30
TD 65536
SOLVENT DMSO
NS 256
DS 4
SWH 18028.846 Hz
FIDRES 0.275098 Hz
AQ 1.8175818 sec
RG 9
DW 27.733 usec
DE 6.00 usec
TE 303.0 K
D1 2.00000000 sec
d11 0.03000000 sec
DELTA 1.89999998 sec
TD0 1
======== CHANNEL f1 ========
NUC1 13C
P1 8.70 usec
PL1 -3.00 dB
SFO1 75.4752953 MHz
======== CHANNEL f2 ========
CPDPRG2 waltz16
NUC2 1H
PCPD2 80.00 usec
PL2 -1.00 dB
PL12 17.00 dB
PL13 21.00 dB
SFO2 300.1312005 MHz
F2 - Processing parameters
SI 32768
SF 75.4677882 MHz
WDW EM
SSB 0
LB 1.00 Hz
GB 0
PC 1.40
200 190 180 170 160 150 140 130 120 110 100 90 80 70 60 50 40 30 20 10 ppm

2h

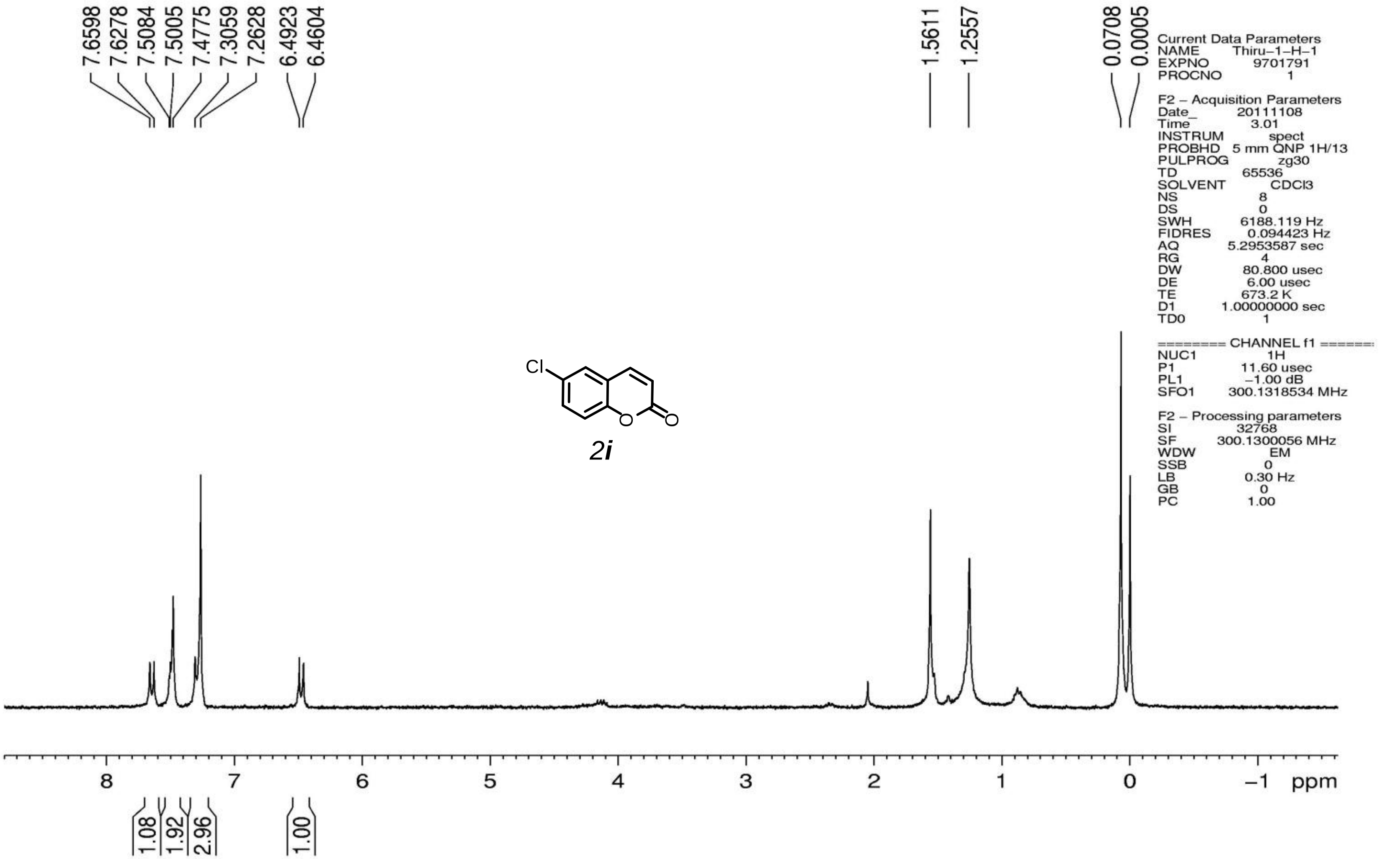

7.6598
7.6278
7.5084
7.5005
7.4775
7.3059
7.2628
6.4923
6.4604
1.5611
1.2557
0.0708
0.0005
Current Data Parameters
NAME Thiru-1-H-1
EXPNO 9701791
PROCNO 1
F2 - Acquisition Parameters
Date_ 20111108
Time 3.01
INSTRUM spect
PROBHD 5 mm QNP 1H/13
PULPROG zg30
TD 65536
SOLVENT CDCl3
NS 8
DS 0
SWH 6188.119 Hz
FIDRES 0.094423 Hz
AQ 5.2953587 sec
RG 4
DW 80.800 usec
DE 6.00 usec
TE 673.2 K
D1 1.00000000 sec
TD0 1
======== CHANNEL f1 ========
NUC1 1H
P1 11.60 usec
PL1 -1.00 dB
SFO1 300.1318534 MHz
F2 - Processing parameters
SI 32768
SF 300.1300056 MHz
WDW EM
SSB 0
LB 0.30 Hz
GB 0
PC 1.00
Cl
O
O
2i
8
7
6
5
4
3
2
1
0
-1
ppm
1.08
1.92
2.96
1.00

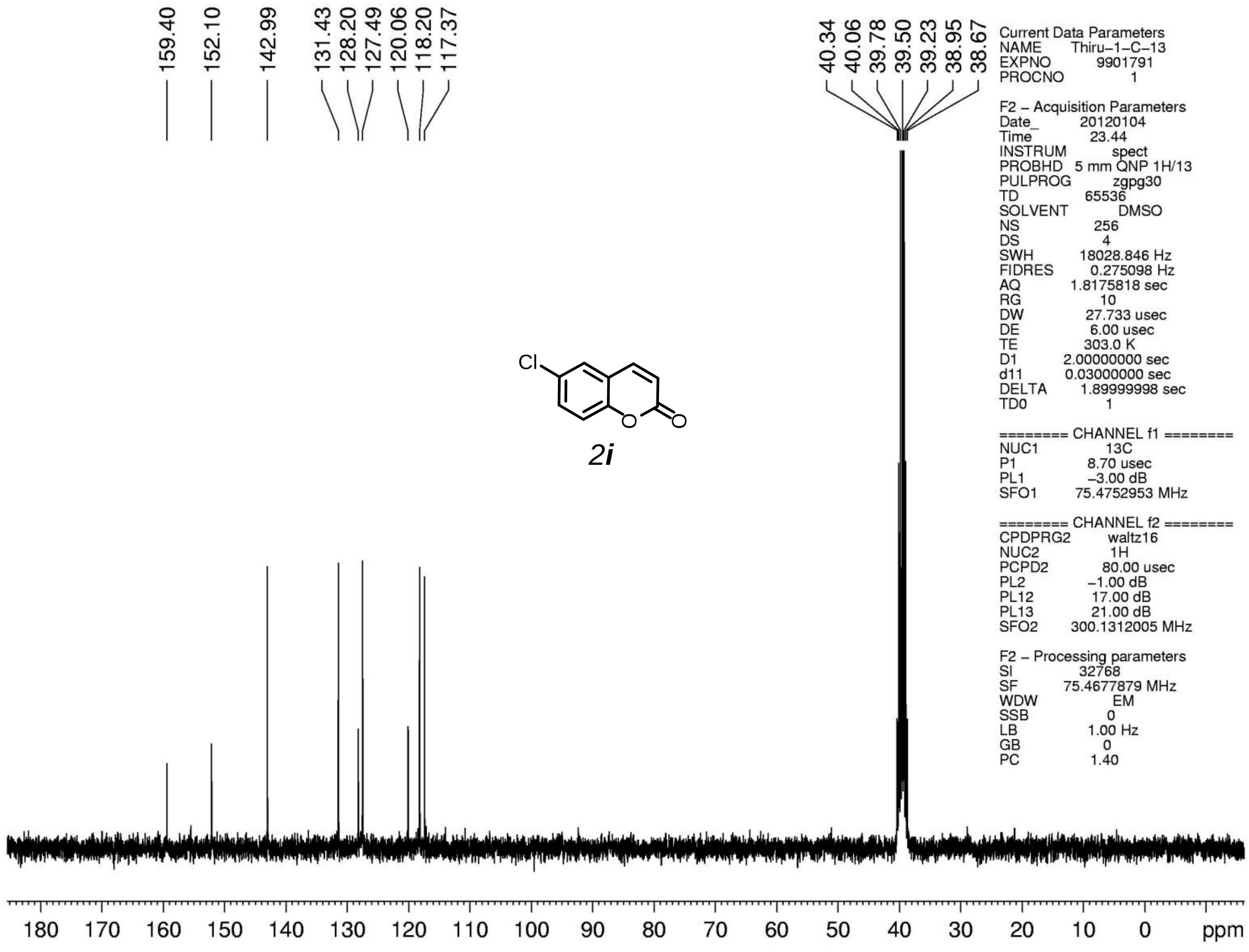
159.40
152.10
142.99
131.43
128.20
127.49
120.06
118.20
117.37
40.34
40.06
39.78
39.50
39.23
38.95
38.67
Cl
O
O
2i
Current Data Parameters
NAME Thiru–1–C–13
EXPNO 9901791
PROCNO 1
F2 – Acquisition Parameters
Date_ 20120104
Time 23.44
INSTRUM spect
PROBHD 5 mm QNP 1H/13
PULPROG zgpg30
TD 65536
SOLVENT DMSO
NS 256
DS 4
SWH 18028.846 Hz
FIDRES 0.275098 Hz
AQ 1.8175818 sec
RG 10
DW 27.733 usec
DE 6.00 usec
TE 303.0 K
D1 2.00000000 sec
d11 0.03000000 sec
DELTA 1.89999998 sec
TD0 1
======== CHANNEL f1 ========
NUC1 13C
P1 8.70 usec
PL1 –3.00 dB
SFO1 75.4752953 MHz
======== CHANNEL f2 ========
CPDPRG2 waltz16
NUC2 1H
PCPD2 80.00 usec
PL2 –1.00 dB
PL12 17.00 dB
PL13 21.00 dB
SFO2 300.1312005 MHz
F2 – Processing parameters
SI 32768
SF 75.4677879 MHz
WDW EM
SSB 0
LB 1.00 Hz
GB 0
PC 1.40
180 170 160 150 140 130 120 110 100 90 80 70 60 50 40 30 20 10 0 ppm

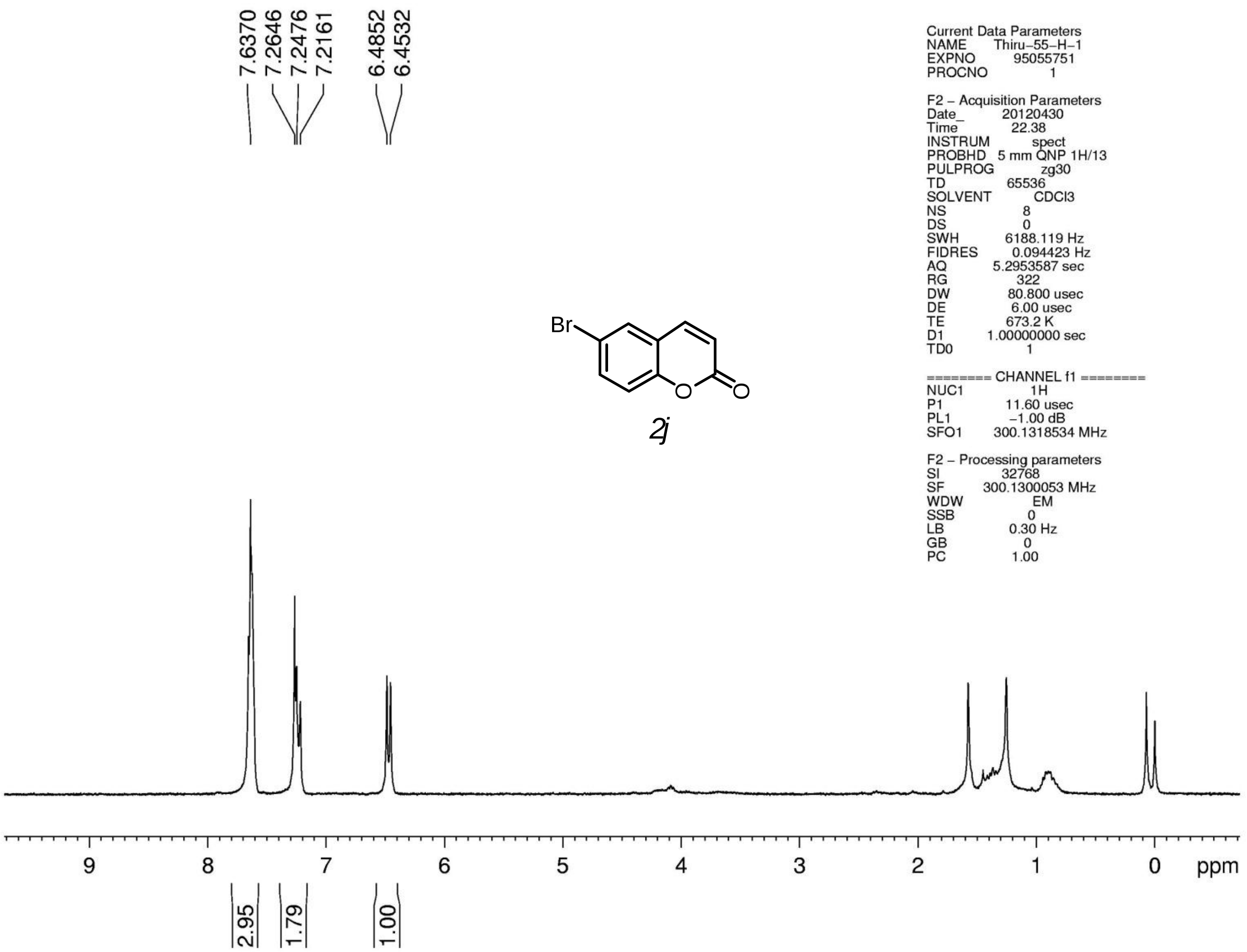
7.6370
7.2646
7.2476
7.2161
6.4852
6.4532
Current Data Parameters
NAME Thiru–55–H–1
EXPNO 95055751
PROCNO 1
F2 – Acquisition Parameters
Date_ 20120430
Time 22.38
INSTRUM spect
PROBHD 5 mm QNP 1H/13
PULPROG zg30
TD 65536
SOLVENT CDCl3
NS 8
DS 0
SWH 6188.119 Hz
FIDRES 0.094423 Hz
AQ 5.2953587 sec
RG 322
DW 80.800 usec
DE 6.00 usec
TE 673.2 K
D1 1.00000000 sec
TD0 1
======== CHANNEL f1 ========
NUC1 1H
P1 11.60 usec
PL1 –1.00 dB
SFO1 300.1318534 MHz
F2 – Processing parameters
SI 32768
SF 300.1300053 MHz
WDW EM
SSB 0
LB 0.30 Hz
GB 0
PC 1.00
Br
O
O
9
8
7
6
5
4
3
2
1
0
ppm
2.95
1.79
1.00

2j

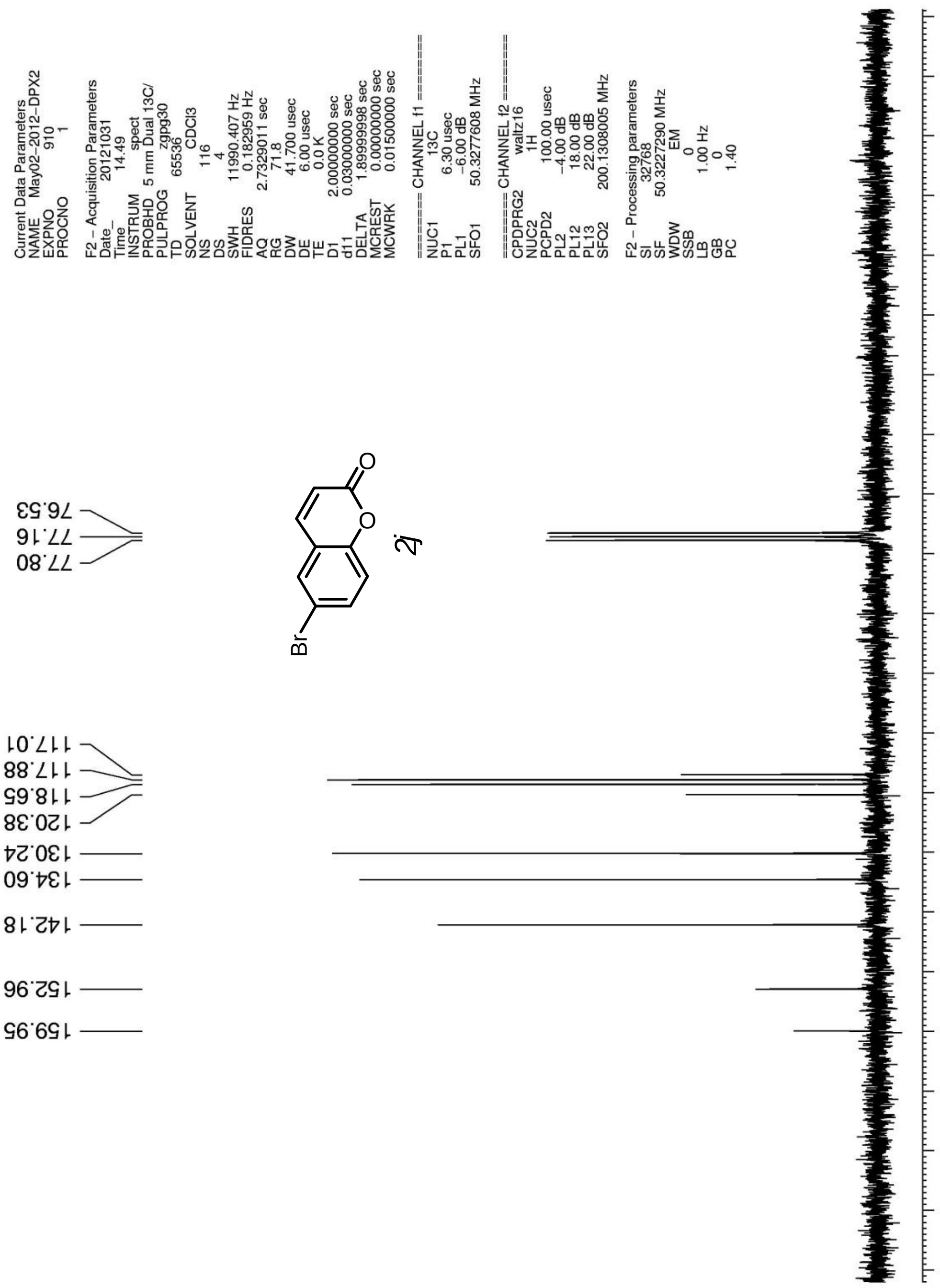

Current Data Parameters
NAME May02-2012-DPX2
EXPNO 910
PROCNO 1
F2 - Acquisition Parameters
Date_ 20121031
Time 14.49
INSTRUM spect
PROBHD 5 mm Dual 13C/
PULPROG zgpg30
TD 65536
SOLVENT CDCl3
NS 116
DS 4
SWH 11990.407 Hz
FIDRES 0.182959 Hz
AQ 2.7329011 sec
RG 71.8
DW 41.700 usec
DE 6.00 usec
TE 0.0 K
D1 2.00000000 sec
d11 0.03000000 sec
DELTA 1.89999998 sec
MCREST 0.00000000 sec
MCWRK 0.01500000 sec
======== CHANNEL f1 ========
NUC1 13C
P1 6.30 usec
PL1 -6.00 dB
SFO1 50.3277608 MHz
======== CHANNEL f2 ========
CPDPRG2 waltz16
NUC2 1H
PCPD2 100.00 usec
PL2 -4.00 dB
PL12 18.00 dB
PL13 22.00 dB
SFO2 200.1308005 MHz
F2 - Processing parameters
SI 32768
SF 50.3227290 MHz
WDW EM
SSB 0
LB 1.00 Hz
GB 0
PC 1.40
159.95
152.96
142.18
134.60
130.24
120.38
118.65
117.88
117.01
77.80
77.16
76.53
Br
O
O
2j
190
180
170
160
150
140
130
120
110
100
90
80
70
60
50
40
30
20
10
0
ppm

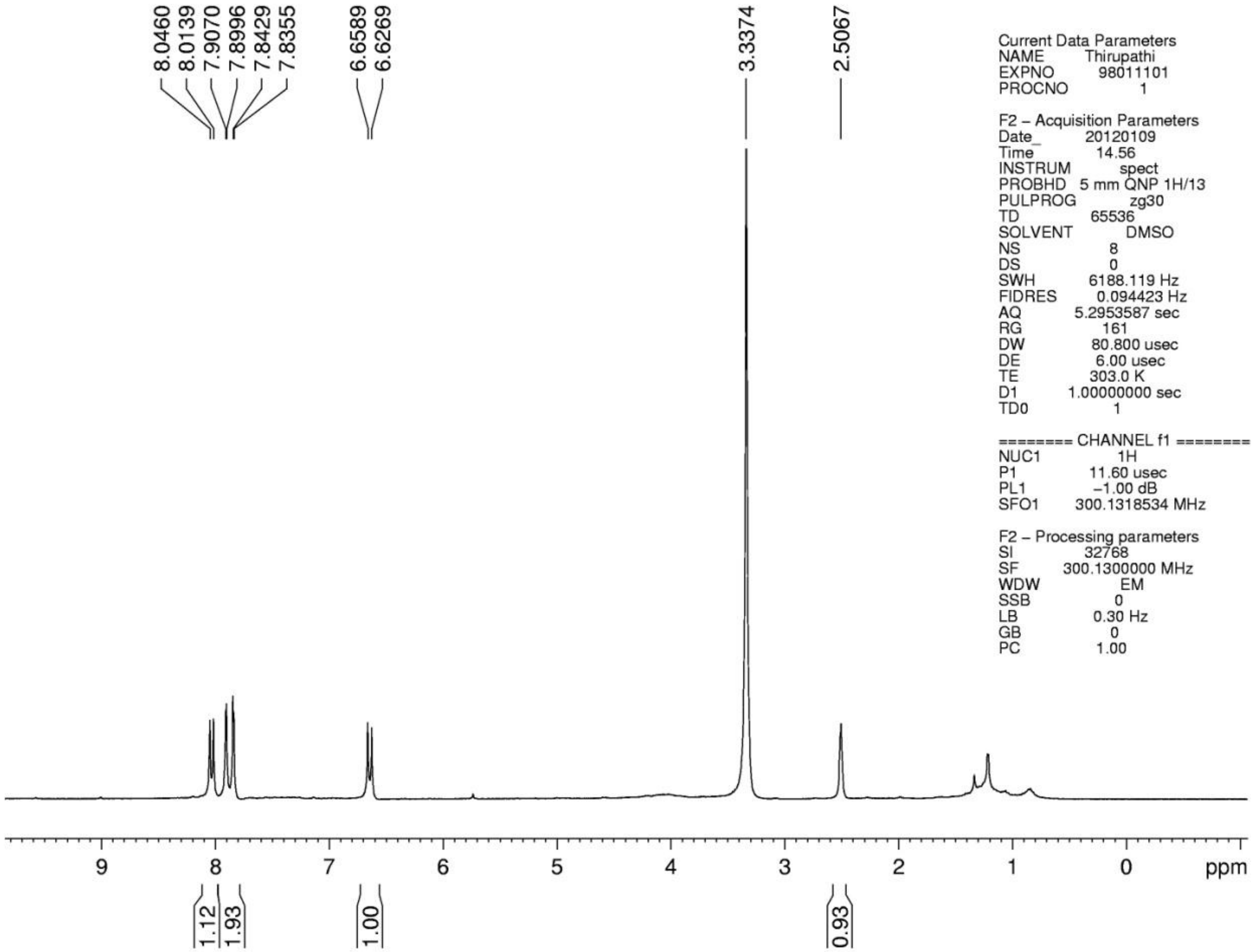
8.0460
8.0139
7.9070
7.8996
7.8429
7.8355
6.6589
6.6269
3.3374
2.5067
Current Data Parameters
NAME Thirupathi
EXPNO 98011101
PROCNO 1
F2 – Acquisition Parameters
Date_ 20120109
Time 14.56
INSTRUM spect
PROBHD 5 mm QNP 1H/13
PULPROG zg30
TD 65536
SOLVENT DMSO
NS 8
DS 0
SWH 6188.119 Hz
FIDRES 0.094423 Hz
AQ 5.2953587 sec
RG 161
DW 80.800 usec
DE 6.00 usec
TE 303.0 K
D1 1.00000000 sec
TD0 1
======== CHANNEL f1 ========
NUC1 1H
P1 11.60 usec
PL1 –1.00 dB
SFO1 300.1318534 MHz
F2 – Processing parameters
SI 32768
SF 300.1300000 MHz
WDW EM
SSB 0
LB 0.30 Hz
GB 0
PC 1.00
9
8
7
6
5
4
3
2
1
0
ppm
1.12
1.93
1.00
0.93

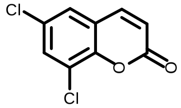
Cl
Cl
O
O

2k

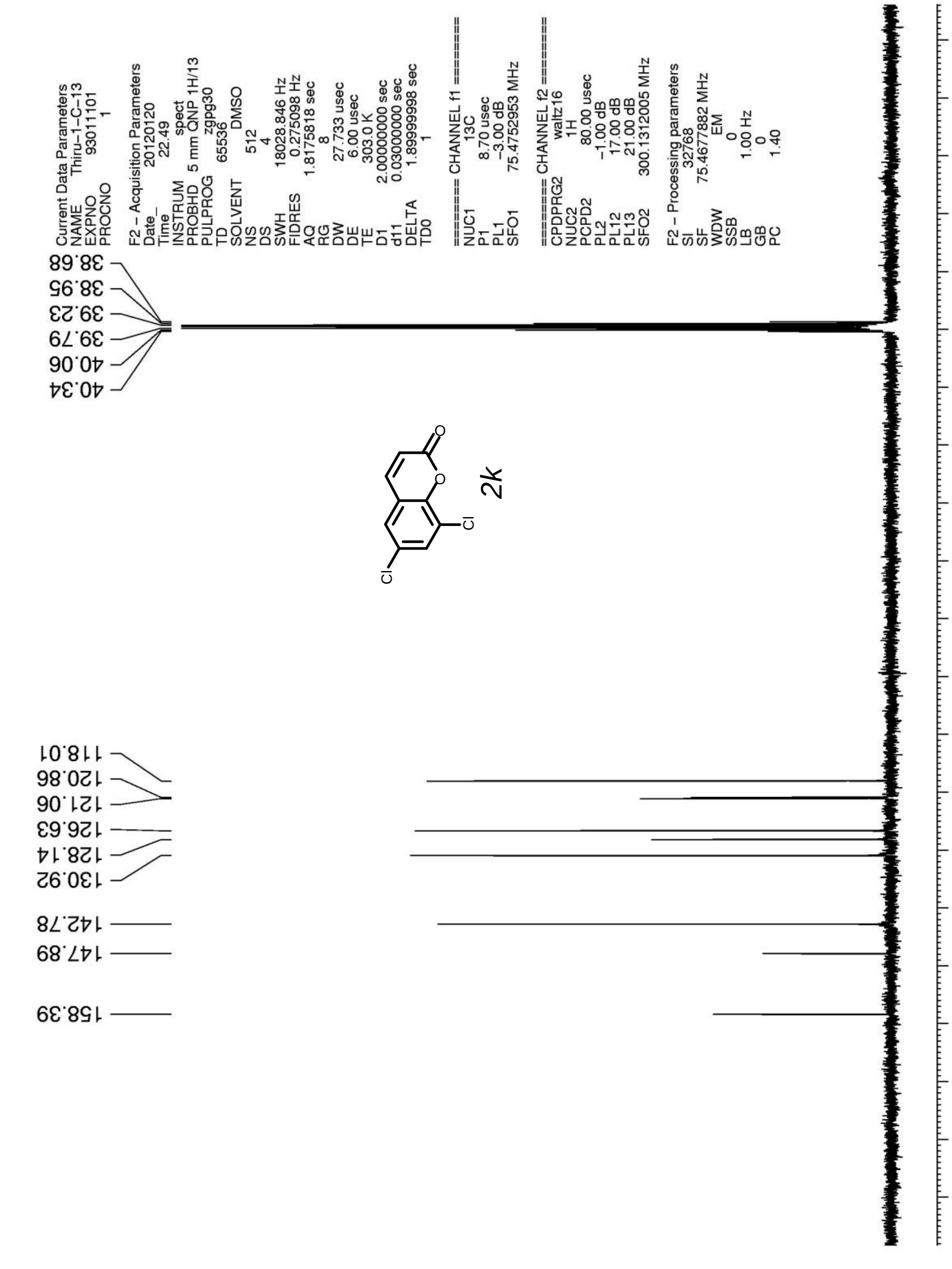

38.68
38.95
39.23
39.79
40.06
40.34
118.01
120.86
121.06
126.63
128.14
130.92
142.78
147.89
158.39
Current Data Parameters
NAME Thiru-1-C-13
EXPNO 93011101
PROCNO 1
F2 – Acquisition Parameters
Date_ 20120120
Time 22.49
INSTRUM spect
PROBHD 5 mm QNP 1H/13
PULPROG zgpg30
TD 65536
SOLVENT DMSO
NS 512
DS 4
SWH 18028.846 Hz
FIDRES 0.275098 Hz
AQ 1.8175818 sec
RG 8
DW 27.733 usec
DE 6.00 usec
TE 303.0 K
D1 2.00000000 sec
d11 0.03000000 sec
DELTA 1.89999998 sec
TD0 1
======== CHANNEL f1 ========
NUC1 13C
P1 8.70 usec
PL1 -3.00 dB
SFO1 75.4752953 MHz
======== CHANNEL f2 ========
CPDPRG2 waltz16
NUC2 1H
PCPD2 80.00 usec
PL2 -1.00 dB
PL12 17.00 dB
PL13 21.00 dB
SFO2 300.1312005 MHz
F2 – Processing parameters
SI 32768
SF 75.4677882 MHz
WDW EM
SSB 0
LB 1.00 Hz
GB 0
PC 1.40
Cl
Cl
O
O
2k
190 180 170 160 150 140 130 120 110 100 90 80 70 60 50 40 30 20 10 0 ppm

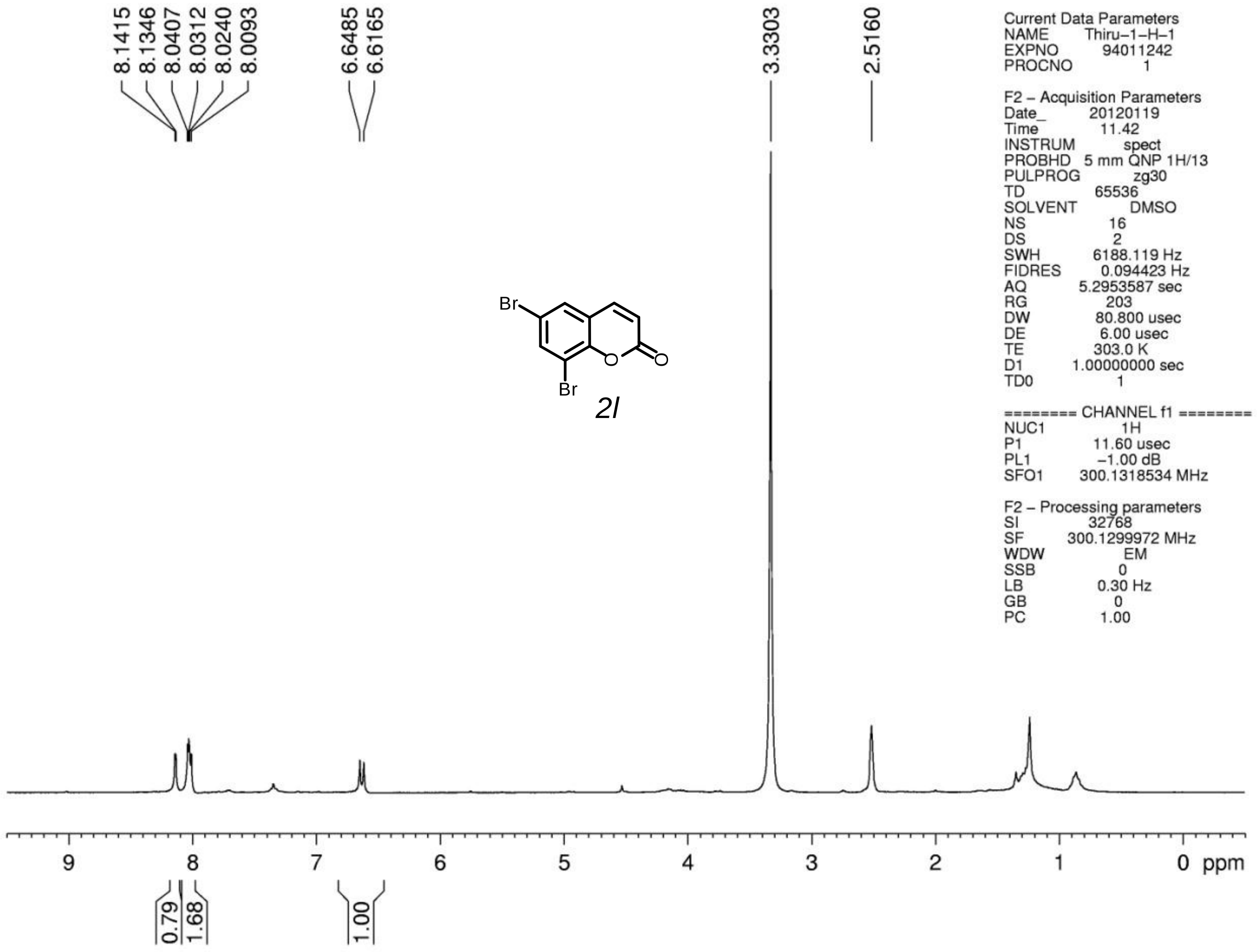
8.1415
8.1346
8.0407
8.0312
8.0240
8.0093
6.6485
6.6165
3.3303
2.5160
Current Data Parameters
NAME Thiru-1-H-1
EXPNO 94011242
PROCNO 1
F2 - Acquisition Parameters
Date_ 20120119
Time 11.42
INSTRUM spect
PROBHD 5 mm QNP 1H/13
PULPROG zg30
TD 65536
SOLVENT DMSO
NS 16
DS 2
SWH 6188.119 Hz
FIDRES 0.094423 Hz
AQ 5.2953587 sec
RG 203
DW 80.800 usec
DE 6.00 usec
TE 303.0 K
D1 1.00000000 sec
TD0 1
======== CHANNEL f1 ========
NUC1 1H
P1 11.60 usec
PL1 -1.00 dB
SFO1 300.1318534 MHz
F2 - Processing parameters
SI 32768
SF 300.1299972 MHz
WDW EM
SSB 0
LB 0.30 Hz
GB 0
PC 1.00
Br
Br
O
O
2l
9
8
7
6
5
4
3
2
1
0 ppm
0.79
1.68
1.00

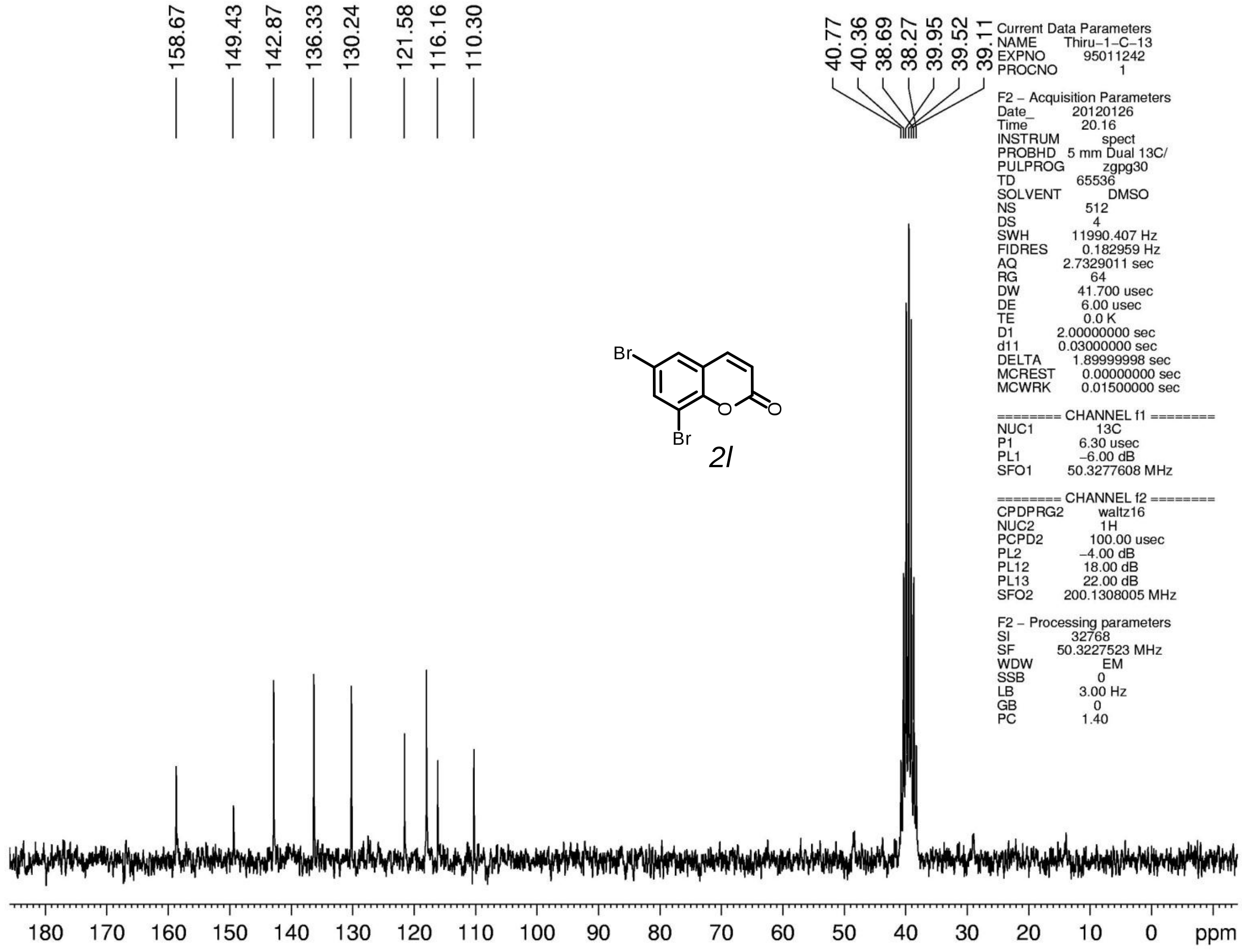
158.67
149.43
142.87
136.33
130.24
121.58
116.16
110.30
40.77
40.36
38.69
38.27
39.95
39.52
39.11
Current Data Parameters
NAME Thiru-1-C-13
EXPNO 95011242
PROCNO 1
F2 - Acquisition Parameters
Date_ 20120126
Time 20.16
INSTRUM spect
PROBHD 5 mm Dual 13C/
PULPROG zgpg30
TD 65536
SOLVENT DMSO
NS 512
DS 4
SWH 11990.407 Hz
FIDRES 0.182959 Hz
AQ 2.7329011 sec
RG 64
DW 41.700 usec
DE 6.00 usec
TE 0.0 K
D1 2.00000000 sec
d11 0.03000000 sec
DELTA 1.89999998 sec
MCREST 0.00000000 sec
MCWRK 0.01500000 sec
======== CHANNEL f1 ========
NUC1 13C
P1 6.30 usec
PL1 -6.00 dB
SFO1 50.3277608 MHz
======== CHANNEL f2 ========
CPDPRG2 waltz16
NUC2 1H
PCPD2 100.00 usec
PL2 -4.00 dB
PL12 18.00 dB
PL13 22.00 dB
SFO2 200.1308005 MHz
F2 - Processing parameters
SI 32768
SF 50.3227523 MHz
WDW EM
SSB 0
LB 3.00 Hz
GB 0
PC 1.40
Br
Br
O
O
2l
180 170 160 150 140 130 120 110 100 90 80 70 60 50 40 30 20 10 0 ppm

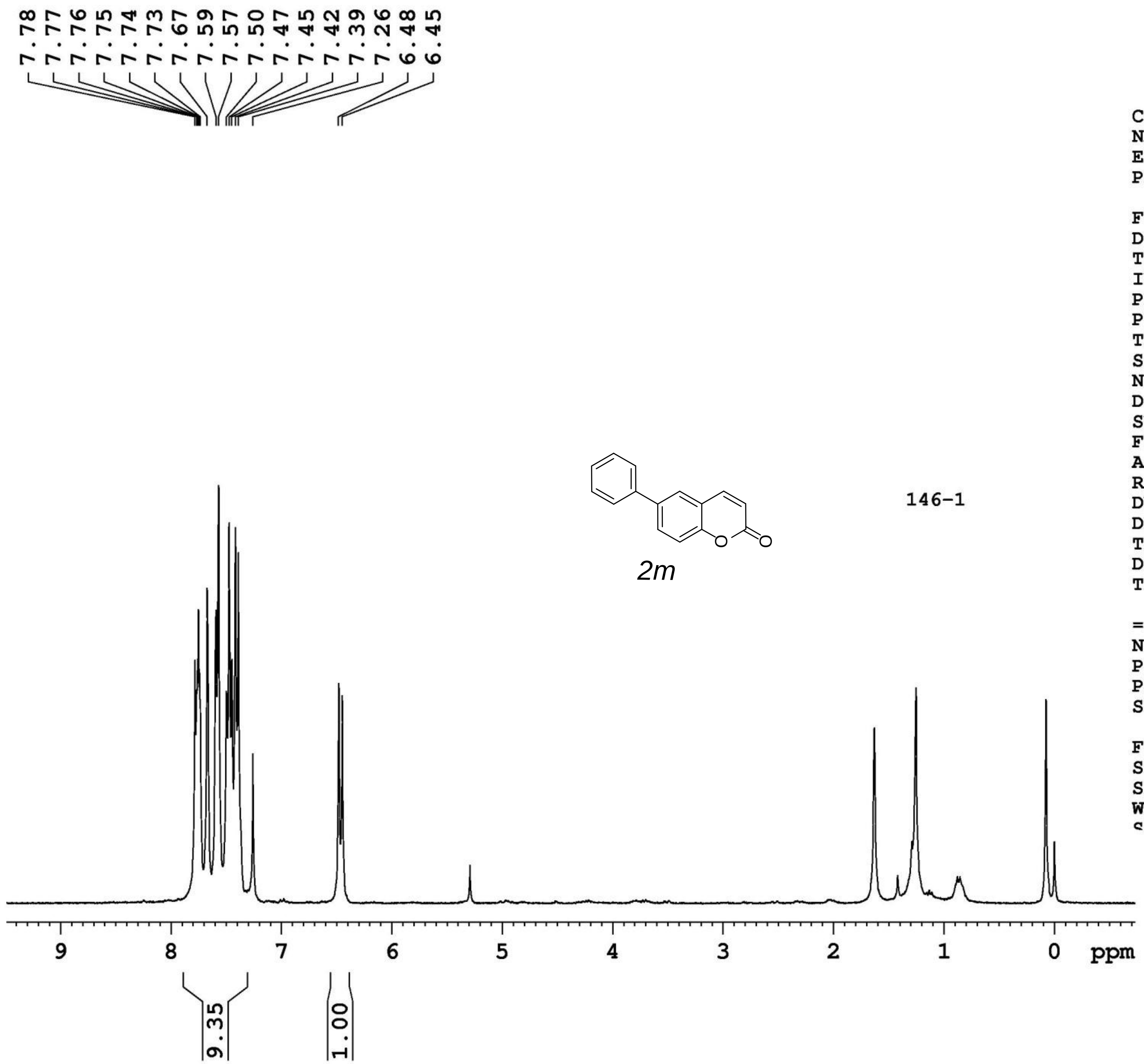


```
Current Data Parameters
NAME     New Folder (8)
EXPNO        970551461
PROCNO               1

F2 - Acquisition Parame
Date_         20120703
Time             14.22
INSTRUM          spect
PROBHD   5 mm QNP 1H/13
PULPROG           zg30
TD               65536
SOLVENT          CDCl3
NS                   8
DS                   0
SWH           6188.119
FIDRES        0.094423
AQ           5.2953587
RG                 203
DW              80.800
DE                6.00
TE               297.6
D1          1.00000000
TD0                  1

======== CHANNEL f1 ===
NUC1                1H
P1               11.60
PL1              -1.00
SFO1       300.1318534

F2 - Processing paramet
SI               32768
SF         300.1300067
WDW                 EM
SSB                  0
```

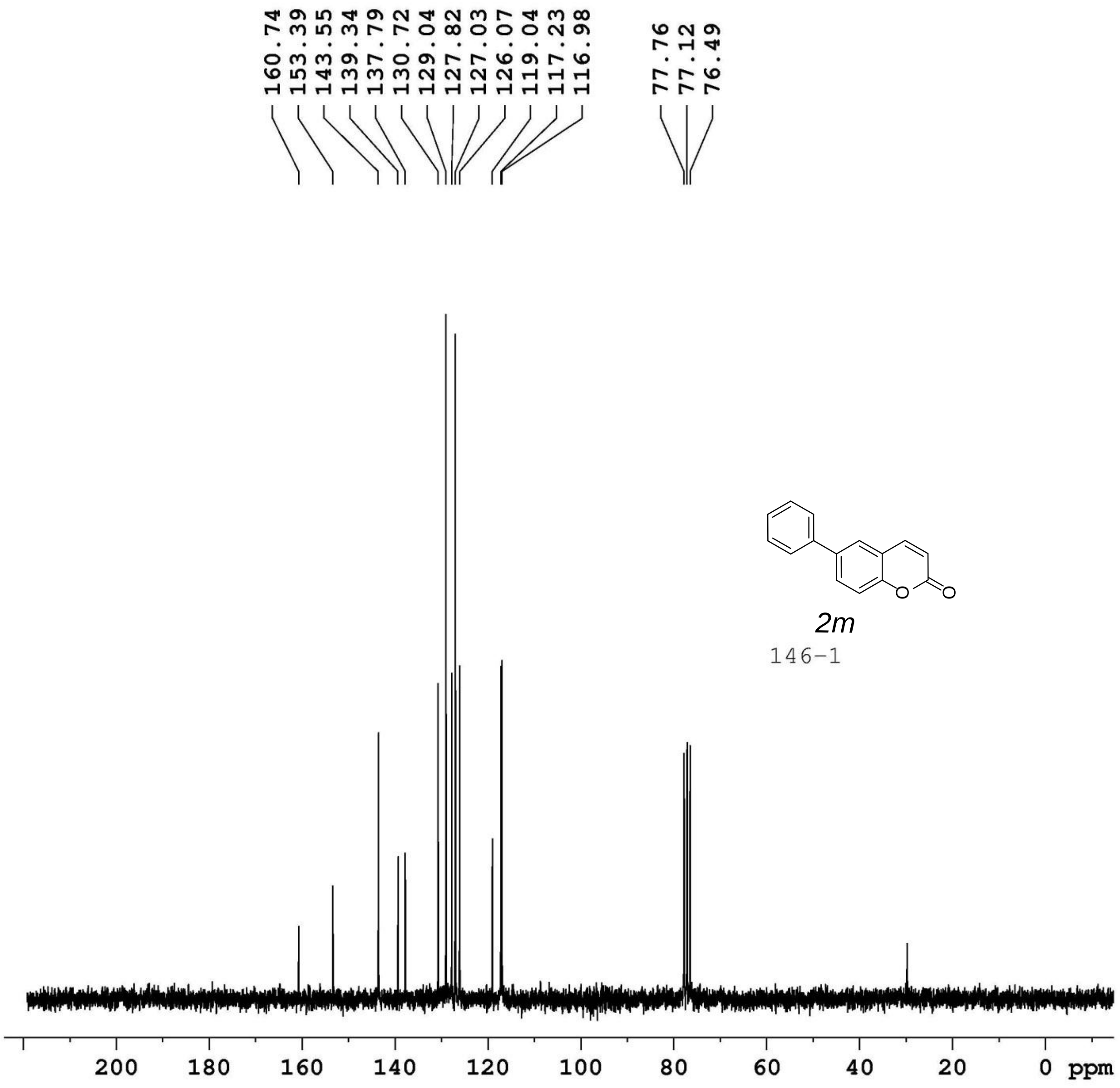

2m

146-1

```
Current Data Paramet[illegible]
NAME     New Folder
EXPNO        910551[illegible]
PROCNO

F2 - Acquisition Par[illegible]
Date_          20120[illegible]
Time              11
INSTRUM           sp[illegible]
PROBHD   5 mm Dual 1[illegible]
PULPROG          zgp[illegible]
TD                65[illegible]
SOLVENT           CD[illegible]
NS                 [illegible]
DS
SWH          11990.[illegible]
FIDRES         0.182[illegible]
AQ            2.7329[illegible]
RG                 4[illegible]
DW               41.[illegible]
DE                 6
TE                 [illegible]
D1          2.000000
d11         0.030000
DELTA       1.899999
MCREST      0.000000
MCWRK       0.015000

======== CHANNEL f1 [illegible]
NUC1               [illegible]
P1                 6
```

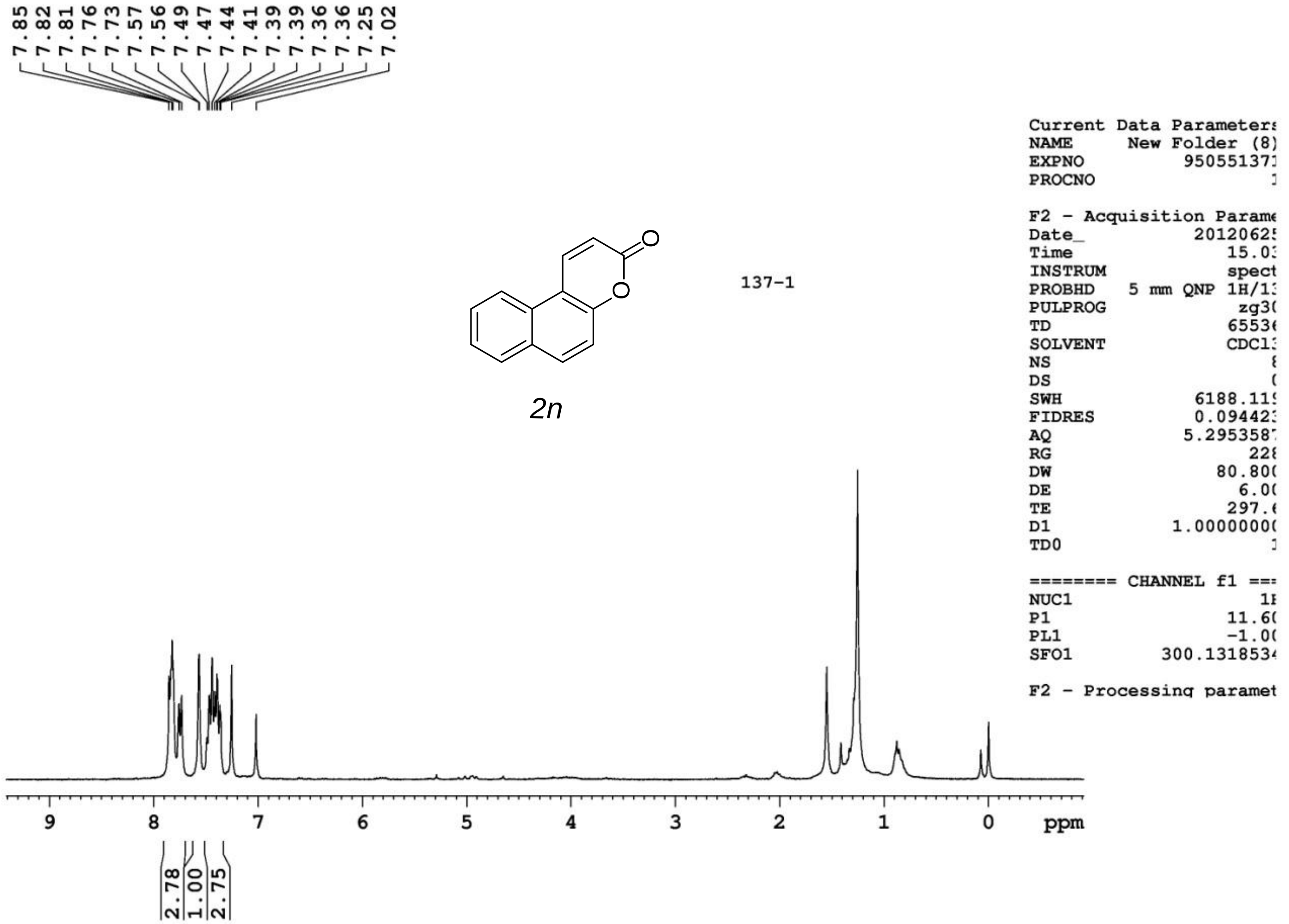
7.85
7.82
7.81
7.76
7.73
7.57
7.56
7.49
7.47
7.44
7.41
7.39
7.39
7.36
7.36
7.25
7.02
2n
137-1
Current Data Parameters
NAME New Folder (8)
EXPNO 950551371
PROCNO 1
F2 - Acquisition Parame
Date_ 2012062
Time 15.03
INSTRUM spect
PROBHD 5 mm QNP 1H/13
PULPROG zg30
TD 65536
SOLVENT CDCl3
NS 8
DS 0
SWH 6188.119
FIDRES 0.094423
AQ 5.2953587
RG 228
DW 80.800
DE 6.00
TE 297.6
D1 1.0000000
TD0 1
======== CHANNEL f1 ===
NUC1 1H
P1 11.60
PL1 -1.00
SFO1 300.1318534
F2 - Processing paramet
9
8
7
6
5
4
3
2
1
0
ppm
2.78
1.00
2.75

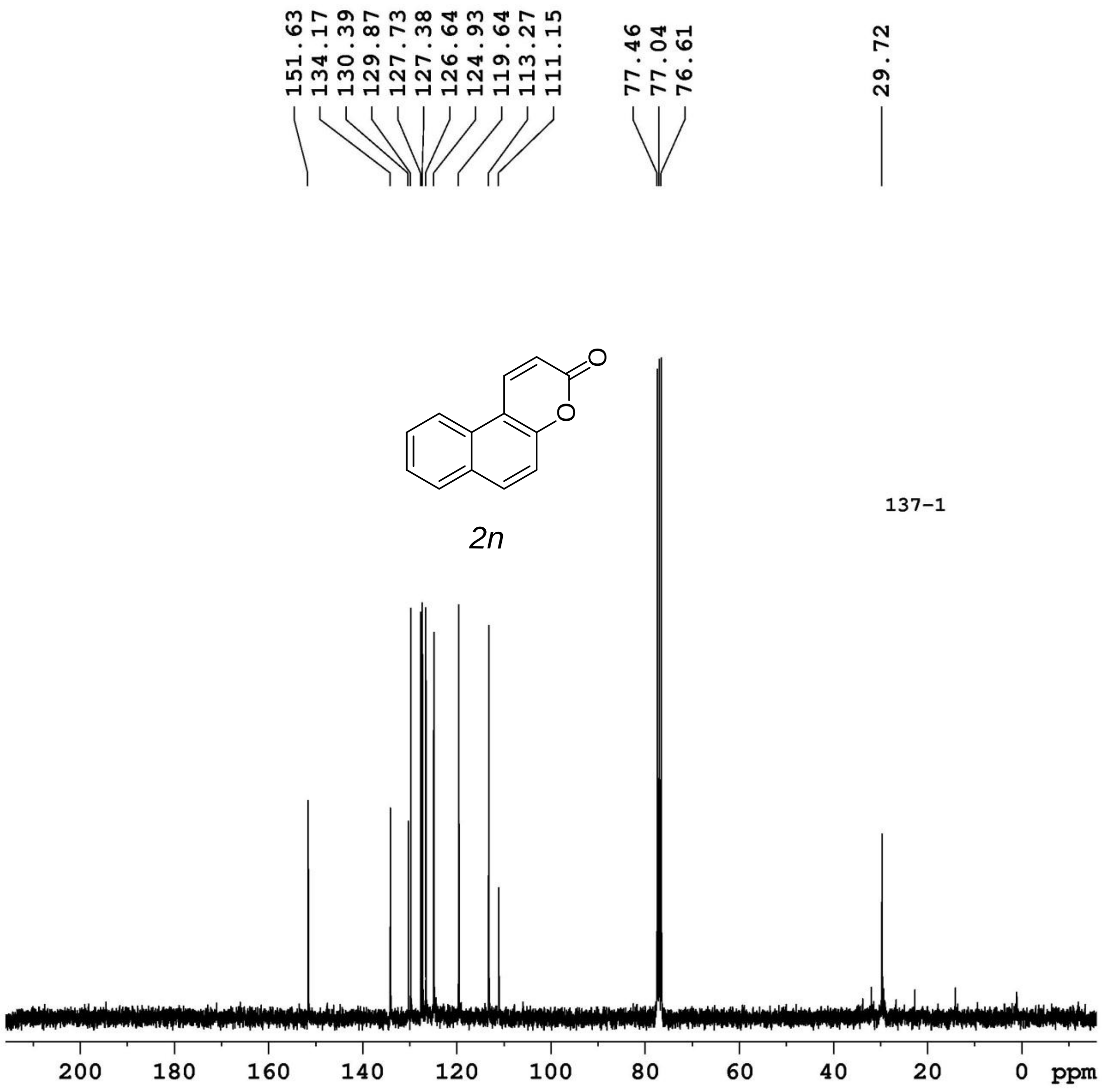


```
Current Data Parameters
NAME      New Folder (8)
EXPNO         1100551371
PROCNO                 1

F2 - Acquisition Paramete
Date_           20120630
Time                7.25
INSTRUM            spect
PROBHD   5 mm QNP 1H/13
PULPROG          zgpg30
TD                 65536
SOLVENT            CDCl3
NS                   512
DS                     4
SWH            18028.846 H
FIDRES          0.275098 H
AQ             1.8175818 s
RG                  12.7
DW                27.733 u
DE                  6.00 u
TE                 303.0 K
D1            2.00000000 s
d11           0.03000000 s
DELTA         1.89999998 s
TD0                    1

======== CHANNEL f1 =====
NUC1                 13C
P1                  8.70 u
PL1                -3.00 d
SFO1         75.4752953 M

======== CHANNEL f2 =====
CPDPRG2          waltz16
NUC2                  1H
PCPD2              80.00 u
```

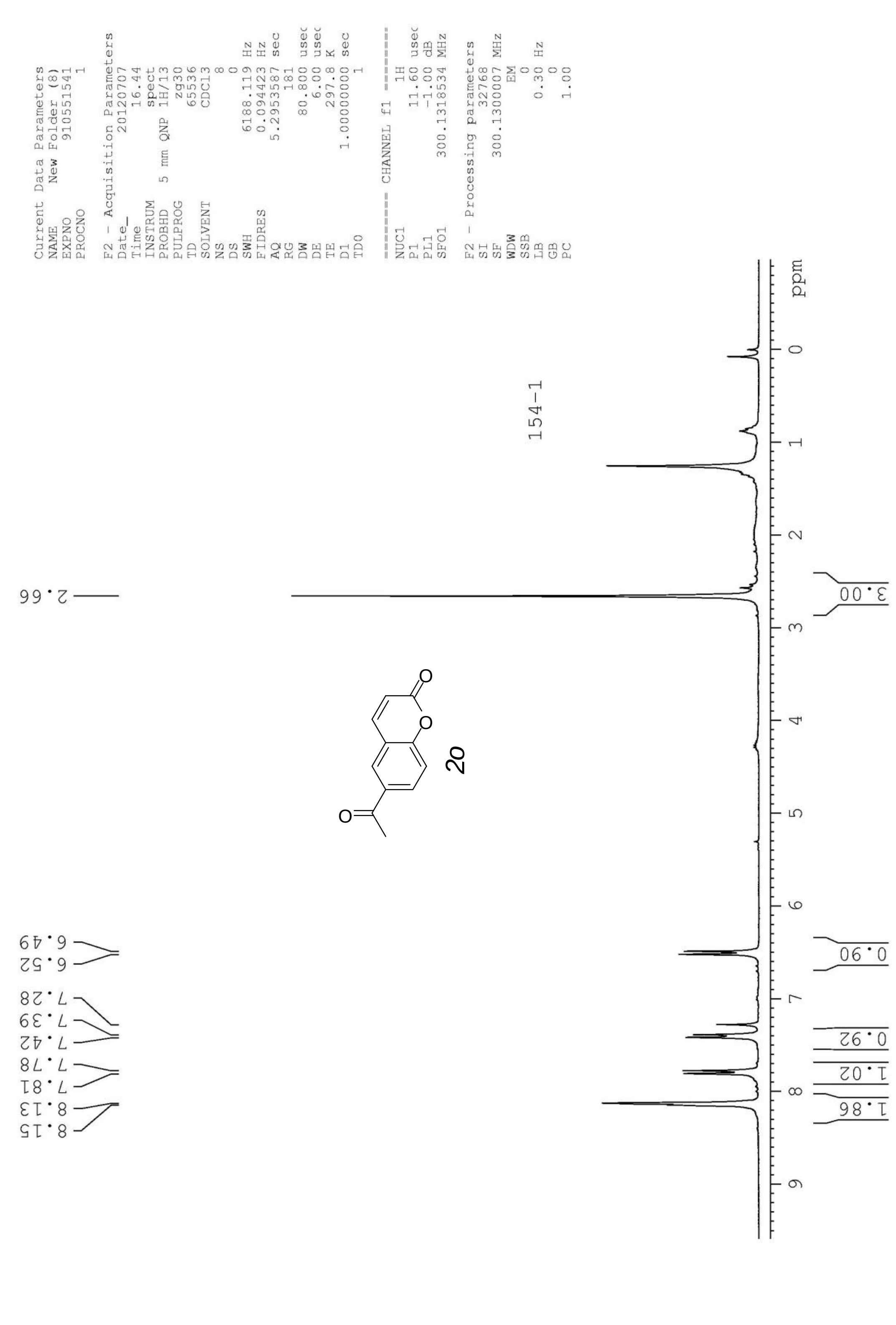
Current Data Parameters
NAME New Folder (8)
EXPNO 910551541
PROCNO 1
F2 - Acquisition Parameters
Date_ 20120707
Time 16.44
INSTRUM spect
PROBHD 5 mm QNP 1H/13
PULPROG zg30
TD 65536
SOLVENT CDCl3
NS 8
DS 0
SWH 6188.119 Hz
FIDRES 0.094423 Hz
AQ 5.2953587 sec
RG 181
DW 80.800 usec
DE 6.00 usec
TE 297.8 K
D1 1.00000000 sec
TD0 1
======== CHANNEL f1 ========
NUC1 1H
P1 11.60 usec
PL1 -1.00 dB
SFO1 300.1318534 MHz
F2 - Processing parameters
SI 32768
SF 300.1300007 MHz
WDW EM
SSB 0
LB 0.30 Hz
GB 0
PC 1.00
154-1
2o
8.15
8.13
7.81
7.78
7.42
7.39
7.28
6.52
6.49
2.66
1.86
1.02
0.92
0.90
3.00
9
8
7
6
5
4
3
2
1
0
ppm
